# Health Care for People Who Are Incarcerated: Teaching Third-Year Medical Students About Rights, Challenges, and Avenues of Advocacy

**DOI:** 10.15766/mep_2374-8265.11464

**Published:** 2024-11-07

**Authors:** Anna-Maria South, Kelsey N. Karnik, Sara Hieneman, Anthony A. Mangino, Michelle Lofwall

**Affiliations:** 1 Assistant Professor, Division of Hospital Medicine, Department of Internal Medicine, and Bell Alcohol and Addictions Scholar, University of Kentucky College of Medicine; 2 Biomedical Data Scientist, Department of Biostatistics, University of Kentucky College of Public Health; 3 Fourth-Year Medical Student, University of Kentucky College of Medicine; 4 Biomedical Data Scientist, Department of Biostatistics, University of Kentucky College of Public Health; 5 Professor, Departments of Behavioral Science and Psychiatry, and Bell Alcohol and Addictions Chair, University of Kentucky College of Medicine

**Keywords:** Incarceration, Medication for Opioid Use Disorder, Patient Advocacy, Health Equity, Law & Medicine, Opioids, Addiction, & Pain, Social Determinants of Health, Substance Abuse/Addiction, Diversity, Equity, Inclusion

## Abstract

**Introduction:**

Incarcerated patients are a vulnerable patient population with unique barriers to health care that physicians in every specialty encounter. Current medical school curricula lack universal education on health care for incarcerated people.

**Methods:**

We developed an interactive workshop to provide third-year medical students at the University of Kentucky with information about delivering care outside of dedicated carceral settings to individuals who are incarcerated. The workshop included education on the demographic characteristics and medical conditions present in these populations along with understanding incarcerated persons’ rights to health care and how to interact with them and the associated jail/prison workforce often accompanying them on medical visits. The workshop also discussed advocacy tools aimed at providing equitable health care for incarcerated people. Our workshop consisted of a 30-minute large-group didactic session providing a basic understanding of the health care needs of incarcerated people, followed by a 30-minute small-group discussion of a *JAMA* Viewpoint article focused on advocacy for equitable health care for this patient population. Workshop effectiveness was assessed with a survey before and after the session.

**Results:**

One hundred and five students (55%) completed a self-assessment before and after the workshop, demonstrating an increase in knowledge and confidence on all but one question, with statistically significant *p* values less than .05.

**Discussion:**

Our 1-hour, interactive workshop for medical students in their clinical years demonstrated significant improvement in students’ knowledge and confidence regarding providing health care to people who are incarcerated.

## Educational Objectives

By the end of this activity, learners will be able to:

1. Describe the right to health care among incarcerated patients.
2. Discuss unique challenges when providing health care for incarcerated patients.
3. Identify avenues for physicians to advocate for equitable health care for incarcerated patients.

## Introduction

The United States incarcerates more people than any other nation.^1,2^ Almost one in three Americans or about 100 million people have a history of involvement with the criminal legal system, and approximately 2.2 million Americans are currently incarcerated at any time.^2^ This translates into approximately 100 million individuals who have to address barriers to accessing health care both during and after incarceration.^2^ People who are incarcerated need medical care, but due to the fragmentation of health care in carceral facilities and at time of release, they often receive health care that is not equitable to the care received by patients who are not incarcerated and on Medicaid (insurance for people who live below the poverty line). Patients often enter the carceral system with a high disease burden. The carceral system compounds preexisting illnesses in addition to contributing to the development of new ones,^3^ resulting in a high disease burden in this vulnerable population, including high rates of substance use disorders and other mental health disorders, and increased complications of chronic medical diseases.^2^ Thus, incarceration is considered a social determinant of health.

People who are incarcerated are entitled to health care under an interpretation of the Eighth Amendment following the Supreme Court case of *Estelle v Gamble* in 1976^4^ that considers withholding health care as cruel and unusual punishment.^1,5^ This, however, does not ensure that patients have access to the standard of care. Most clinicians will care for a patient who is currently or has been incarcerated. Yet access to health care for people who are incarcerated remains poor (and inequitable, e.g., with limited access to medication for opioid use disorder [MOUD] or frequent breaches in patient privacy) in current medical practice; thus, it is critical for physicians to learn about the rights of incarcerated persons to health care.^6^

Health care for incarcerated people is not a part of the current standard medical training, and few medical schools have dedicated lectures to discuss the nuances of health care for incarcerated people.^7^ Few carceral systems have partnered with academic medical centers in an attempt to close the gap between medical care provided in and out of carceral facilities by adequately training medical students and residents how to provide equitable health care for people who are incarcerated.^8^ The University of Texas Medical Branch at Galveston (UTMB) is trying to bridge this gap. For example, 80% of health care for the Texas Department of Criminal Justice is provided by UTMB, and almost every medical student there is involved in care of the incarcerated.^9^ Yet a survey at UTMB reveals that even in a system with a close relationship between clinicians and officials of the carceral facility, students report institutional obstacles to providing the standard of care and other disparities when caring for this population.^8^ While health care delivery remains inequitable, educating students results in their recognition of this inequity and the potential to advocate for change.

Previous publications in *MedEdPORTAL* addressing education in the care of people who are incarcerated reflect elective opportunities for students in preclinical years or as one part of a broader curriculum addressing care for vulnerable populations.^9–11^ While these educational modules are extremely valuable, we add to them by providing data from a workshop entitled The Basics of Health Care for Incarcerated People. Our workshop is unique as it is a required part of the curriculum at the University of Kentucky (UK) that presents material for students applying to all different medical specialties and focuses solely on incarcerated patients and their rights. One of the previously published resources is specifically about female patients only,^11^ and none of them focus on advocacy for treatment of substance use disorder. We assessed the effectiveness of our workshop with a survey completed by third-year medical students before and after the didactic session.

## Methods

### Workshop Development

Our workshop was included in a required third-year longitudinal class that students completed during assigned breaks from their clinical duties in their first clerkship year and that taught overarching themes relevant to all medical specialties. We chose to include the workshop in this course as the course was required for all students, crossed specialties, and took place at a time when students first had interactions with patients. The lecture took place in October, after the students had gained some comfort with patient interactions and participating in clerkships but early enough so they could apply the content for the remainder of the academic year and their careers.

The workshop evolved over the course of 3 years using Kern's six-step approach to curriculum development^12^ and has been part of the mandatory curriculum at UK since 2021. The need for the workshop was discovered by one of the authors (Anna-Maria South) during bedside rounds in 2020 because medical students lacked knowledge on health care for incarcerated people. We identified this topic as absent in the curriculum. During the workshop's first iteration in 2021, we taught using an interactive case discussion only in a large-group format without providing basic background information about the unique rights of incarcerated patients. During written course evaluations after the session, students provided feedback that the workshop was important and presented novel content, as well as requesting more discussion of important background concepts. This prompted us to develop specific learning objectives using Bloom's taxonomy.^13^

Our goal was for students to understand the rights of incarcerated people as applicable to health care and the unique challenges resulting from their incarcerated status. During the second iteration in 2022, we developed an interactive didactic session to teach the required clinical background (Appendix A), followed by a small-group discussion. Our implementation success in 2022 was due in part to the support of the course directors and faculty who had served as small-group facilitators in 2021, and we were fortunate to have facilitators who were faculty across different specialties familiar with providing health care for incarcerated patients and able to lead discussions on sensitive topics in a safe learning space. After this 2022 curricular revision, students self-reported increased knowledge in the postlecture assessment (we did not distribute a prelecture assessment). During the small-group discussions, students expressed a desire to take a more prominent advocate role in patient care. Returning to Bloom's taxonomy,^13^ this prompted us to create content for students to apply the learned knowledge. Specifically, we included a peer-reviewed article^14^ focusing on advocacy for health care for incarcerated people for discussion in the 2023 small-group session. Our goal was to teach students basic principles of health care for incarcerated people that would be applicable to all specialties. The 2023 workshop iteration is discussed in this publication. UK's Institutional Review Board reviewed the workshop and evaluation materials and granted expedited approval.

### Workshop Overview

The workshop agenda was outlined in the facilitator guide (Appendix B). The instructional method was a 30-minute interactive large-group didactic session (Appendix A) with 189 students at three different sites (one in-person group, two groups via Zoom simultaneously) that employed the think-pair-share method (where students first consider a question on their own and then discuss with a partner or a group of peers) to establish basic foundational knowledge about health care for people who are incarcerated. Students were not required to have any baseline knowledge prior to attending the session. The session started with the presurvey assessment (3–5 minutes; Appendix C). During the PowerPoint didactic session (25 minutes), we discussed a fictional case of an incarcerated patient needing a surgical procedure to highlight that incarcerated patients can make their own medical decisions and provide informed consent, that they have a right to privacy under the Health Insurance Portability and Accountability Act (HIPAA),^15^ and that shackles can be removed if safe for the clinician and necessary for medical care. We provided an overview of current incarceration status in the United States with the highest rates disproportionally affecting people of color, of lower socioeconomic status, or with a history of substance use disorder.

During the interactive discussion, we compared and contrasted how people who are incarcerated access health care versus people in the community. We discussed common problems such as having no access to a second opinion; fragmentation of medical care during transfer between carceral facilities or after release; often inadequate screening and treatment for substance use disorder, including decreased access to MOUD; increased risk of infectious disease; and challenges with reintegration after release from jail/prison. We made students aware that mortality was increased after incarceration, explaining that overdose was the number one cause of death, which could be addressed through providing access to evidence-base treatment with mortality-reducing MOUD. We review the mechanism of action and benefits of the three Food and Drug Administration–approved MOUDs. We presented a study among surgical residents to highlight how all specialties care for patients who are incarcerated, then emphasized some general guidelines for inpatient care for incarcerated patients to reinforce discussion points (consent, privacy, [un]shackling, and decision-making) from the beginning of the workshop. At the end of the interactive lecture session, students had an opportunity to ask questions before dividing into small groups (approximately eight students per group) for a 30-minute discussion of the lecture and a peer-reviewed article.^14^ Faculty members served as preceptors to guide the discussion using the facilitator guide (Appendix B, part 2). The session concluded with a postlecture assessment (3–5 minutes; Appendix D). The pre- and postassessment each had five questions focusing on knowledge and confidence in providing health care for incarcerated patients. While participation in the workshop was a mandatory part of the curriculum, survey completion was voluntary.

### Workshop Materials

The workshop took place in a large lecture hall, and the small-group discussions took place in adjacent smaller classrooms. Our institution has three campuses. The lecturer presented the lecture at the site with the largest student body, and the other two campuses gathered in a classroom in their locations and joined via Zoom. We displayed the PowerPoint slides for the interactive didactic lecture on a large projector wall that was also visible via Zoom at the other two locations. The *JAMA* Viewpoint article^14^ for the small-group discussion was available online for students and faculty to print or read on their personal electronic devices. The assessment surveys were accessible via QR code on the PowerPoint slides before and after the lecture, and students used their personal devices (cell phone, laptop) to complete the surveys.

### Training and Standardization

To standardize training of the facilitators for the small-group discussions across the four campuses of our institution, we created a facilitator guide (Appendix B). We trained all facilitators during a 15-minute session on the key points of the lecture, the article, and the facilitator guide. The facilitators were invited to join the interactive didactic part of the workshop. (If a live facilitator training is not possible, a training session could be recorded for facilitators to watch in an asynchronous fashion.)

### Analysis

To assess whether our workshop increased students’ knowledge and perception of importance of health care for incarcerated people, we analyzed the pre- and postworkshop surveys that the students completed. After the session, we paired pre- and postworkshop student responses by utilizing unique identifiers the students had generated using a letter of their choosing and four numbers. We analyzed the matched responses using asymptotic McNemar tests (the multicategorical extension of the standard McNemar test for paired binary data). The asymptotic McNemar test allowed for the analysis of the categorical responses in our paired data (e.g., true/false/unsure with unsure counted as the incorrect answer choice). This extension enabled us to make meaningful pre- and postworkshop comparisons on the matched pairs outcomes for questions with more than two categories, where data were sparse across cells. We excluded students from the analysis if they did not complete both surveys or did not have a unique identifier we were able to match. A *p* value less than < .05 was considered significant change.

## Results

In 2023, 189 third-year medical students attended this mandatory 1-hour workshop. One hundred and five of them completed both the pre- and postsession survey (Appendices C and D) and were able to be connected via a unique identifier.

The students who completed both surveys self-identified as 69% female, 100% cisgender, 90% White, and 3% Hispanic (Table 1). Prior to the workshop, only 11% reported feeling they had received adequate training on providing health care for people who are incarcerated (Table 2). After completing the workshop, 98% agreed or strongly agreed that the lecture significantly increased their knowledge about providing health care for people who are incarcerated (Table 2).

**Table 1.**
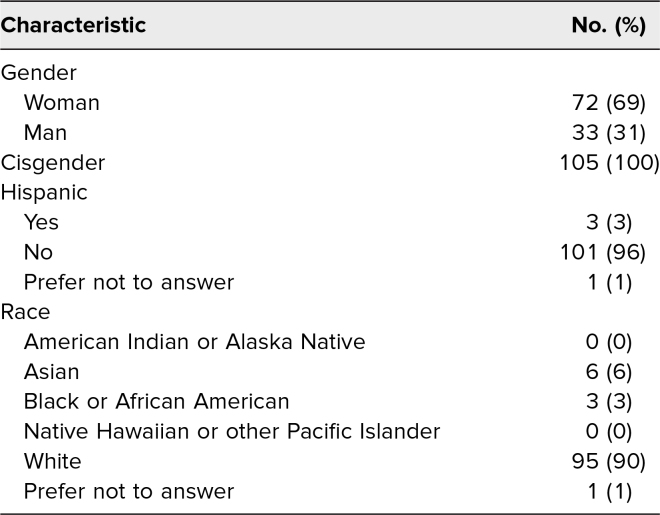
Participant Demographics (*N* = 105)

**Table 2.**
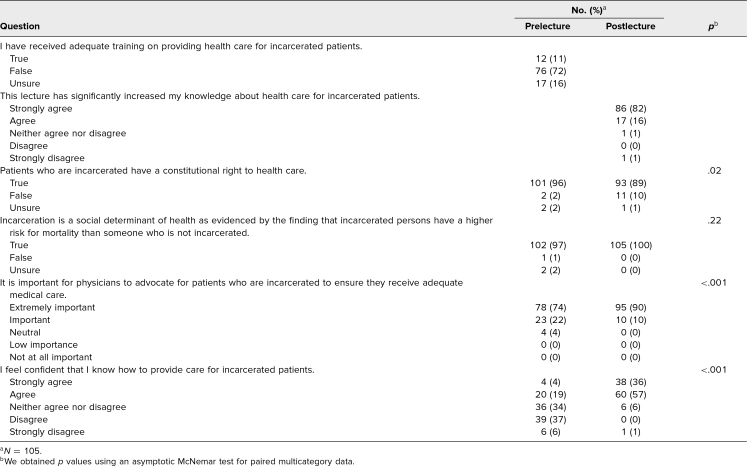
Survey Questions Before and After Workshop

Table 2 also describes the changes observed from pre- to postsurvey for the four questions included in both surveys. The students’ perception of the importance of the role physicians play as health care advocates for patients who are incarcerated changed significantly (*p* < .001): On the presurvey, 74% said the role was extremely important, 22% important, and 4% neutral. These changed on the postsurvey to 90%, 10%, and 0%, respectively. This finding indicates a favorable shift toward advocacy for equitable health care for incarcerated people following the workshop. The largest shift occurred in the students’ perception of their confidence in providing health care for incarcerated people. On the presurvey, a minority of students strongly agreed (*n* = 4, 4%) or agreed (*n* = 20, 19%) in contrast to neither agreeing nor disagreeing (*n* = 36, 34%), disagreeing (*n* = 39, 37%) or strongly disagreeing (*n* = 6, 6%). On the postsurvey, students’ perception shifted favorably, with statistically significant results (*p* < .001), with the majority of students agreeing (*n* = 60, 57%) or strongly agreeing (*n* = 38, 36%) compared to the minority who neither agreed nor disagreed (*n* = 6, 6%), none who disagreed, and only one outlier who strongly disagreed (1%). One question assessed students’ knowledge of whether incarceration was a social determinant of health. This was the only question that did not have a significant change, with a *p* value of .22, as 97% of students correctly answered true during the presurvey, with only one student answering false and two unsure. After the lecture, 100% of students answered correctly. Surprisingly, there was a significant decrease in knowledge on the question about incarcerated persons’ right to health care; thus, we will be rephrasing this question to clarify that the Eighth Amendment has been interpreted by the Supreme Court such that withholding health care is considered cruel and unusual punishment, a concept emphasized heavily during the didactic session. However, the lecture also emphasized that this did not ensure that people who are incarcerated received equitable health care.

## Discussion

Our workshop is a valuable contribution to medical education. It is an interactive discussion that provides baseline knowledge about the rights of incarcerated people to health care as well as a potential framework for commonly encountered clinical scenarios, such as ensuring patient privacy in compliance with HIPAA,^15^ establishing a decision-maker process should a patient not be able to make their own decisions, considerations for removing shackles in the clinical setting, and establishing appropriate follow-up for people who are incarcerated. In addition, our workshop focuses on the chronic medical disease of opioid use disorder (OUD) and educates students on the health care rights that people with OUD have, as withholding MOUD is a violation of the Americans with Disabilities Act.^14,16,17^ Our survey assessment results highlight that the workshop significantly increased students’ knowledge about patients who are incarcerated as well as about advocating for and providing equitable health care to this vulnerable population.

Our workshop has several strengths. It is taught across three medical school sites (both urban and rural) at one large state university health care system institution using a hybrid in vivo/virtual modality to a group of medical students in their clinical years who will eventually graduate and enter a variety of different specialties. Therefore, examples from surgical and medical fields are used to help frame discussion. In addition, the workshop provides medical students with specific advocacy tools to increase equitable health care for patients who are incarcerated. The workshop also reviews all three forms of MOUD and highlights why patients with OUD are a particularly vulnerable patient group when they are incarcerated. In addition, the workshop highlights different social determinants of health such as race and socioeconomic status that are overrepresented in people who are incarcerated. This strengthens students’ understanding of the role of physicians as patient advocates who need to ensure that patients have access to the standard of care and that root causes of health problems are addressed.^18^ *Estelle v Gamble* only guarantees that patients who are incarcerated receive some sort of health care, not that this care be equivalent to health care for people who are not incarcerated.^4^ Therefore, stronger advocacy by physicians is necessary to ensure that all health care needs for this vulnerable patient population are met. This contradiction appears to still be unclear to students and can be further explored in the small-group sessions. We advise emphasizing in the didactic that there is a constitutional right to health care. The speaker may want to do a quick poll of the class after making this point to ensure it has been understood—for example, “So, please raise your hand if you believe it is true that health care is right in the Constitution.”

To address challenges encountered, our workshop underwent several edits. Specifically, we first assumed our students had more background knowledge about health care rights among incarcerated persons and would be comfortable talking in a large group about prisoners, which was incorrect. Students were not able to participate due to not knowing the health care rights of incarcerated patients and appeared uncomfortable speaking in a large-group setting about this sensitive topic, so we introduced more background on constitutional health care rights and small-group settings to address these challenges.

One limitation of the workshop is the number of trained faculty members required for the small-group session, though this can be modified. For institutions that may be challenged to deliver this session as a result of a limited number of facilitators, we offer the following potential adaptations: (1) Students could be trained as facilitators, (2) the small-group sessions could take place in consecutive sessions, (3) the group size could be increased, or (4) the students could break into small groups without a facilitator and discuss the questions independently amongst themselves and then report back to the large group. While students were required to attend the didactic, the surveys were voluntary and those completing the surveys were all cisgender and predominantly White and non-Hispanic. Therefore, our survey might not be representative of our entire student body. This is a brief workshop, with the postsurvey administered immediately after the session, so we have been unable to assess for sustained change in confidence or knowledge. In addition, we did not have a control group, so it is unknown if students’ knowledge would have increased without the lecture. The survey was conducted at a single academic institution within a state highly affected by the opioid epidemic, and results might differ in other regions of the country or at other institutions. In addition, the posttest was conducted after the didactic session and did not capture changes resulting after the small-group sessions, a limitation that, going forward, can be addressed by administering the posttest after the small-group session. In future iterations of the workshop, the survey will be modified to feature more questions, including questions asking if students have personal history or history of a family member or friend with incarceration and questions not limited to confidence but also concerning knowledge and intention to change behavior. Our workshop did not include people with lived experience with incarceration as facilitators. Including facilitators with lived experience would increase students’ understanding of incarceration and the difficulties in accessing health care while incarcerated. Facilitators with lived experience could be recruited from the community for future iterations of the workshop. Also, future evaluations should include a posttest several months after the lecture to assess for sustained knowledge change.

In summary, students’ knowledge and confidence level around advocating for and providing medical care to people who are incarcerated significantly increased after our workshop. The session focuses on teaching basic knowledge about health care for people who are incarcerated and provides advocacy strategies to medical students in their clinical years.

## Appendices


Basics of Health Care for Incarcerated Patients.pptxFacilitator Guide.docxPretraining Session Evaluation.docxPosttraining Session Evaluation.docx
*All appendices are peer reviewed as integral parts of the Original Publication.*
