# Supplementary material for: Health Care for People Who Are Incarcerated: Teaching Third-Year Medical Students About Rights, Challenges, and Avenues of Advocacy
Source: MedEdPORTAL. 2024 Nov 7;20:11464. doi: 10.15766/mep_2374-8265.11464 (PMC11540842; doi:10.15766/mep_2374-8265.11464)
Supplement: Supplementary file 1 — Basics of Health Care for Incarcerated Patients.pptxFacilitator Guide.docxPretraining Session Evaluation.docxPosttraining Session Evaluation.docx [file mep_2374-8265.11464-s001.zip › A. Basics of Health Care for Incarcerated Patients.pptx]

## Slide 1
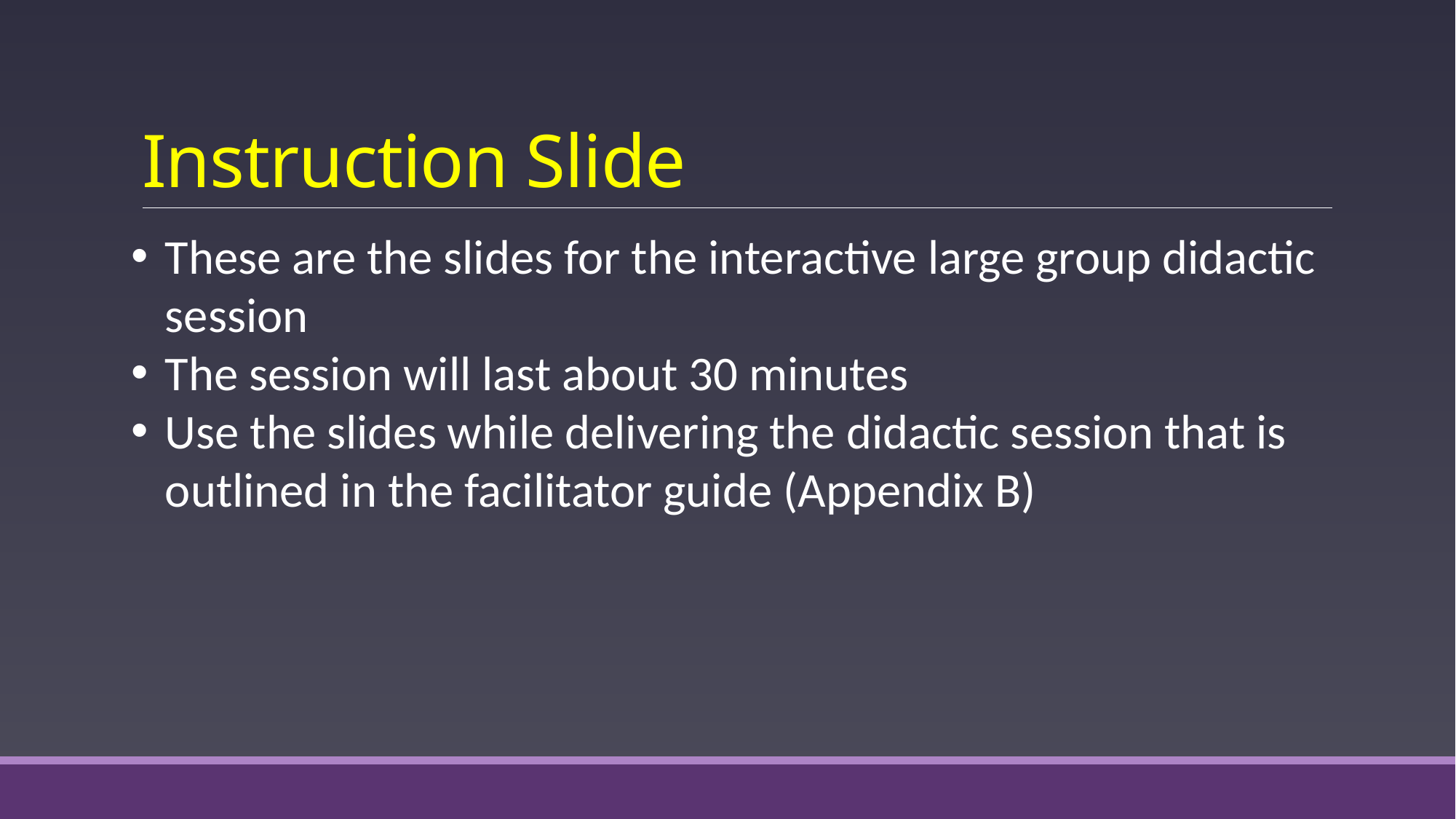

# Instruction Slide
These are the slides for the interactive large group didactic session
The session will last about 30 minutes
Use the slides while delivering the didactic session that is outlined in the facilitator guide (Appendix B)

## Slide 2
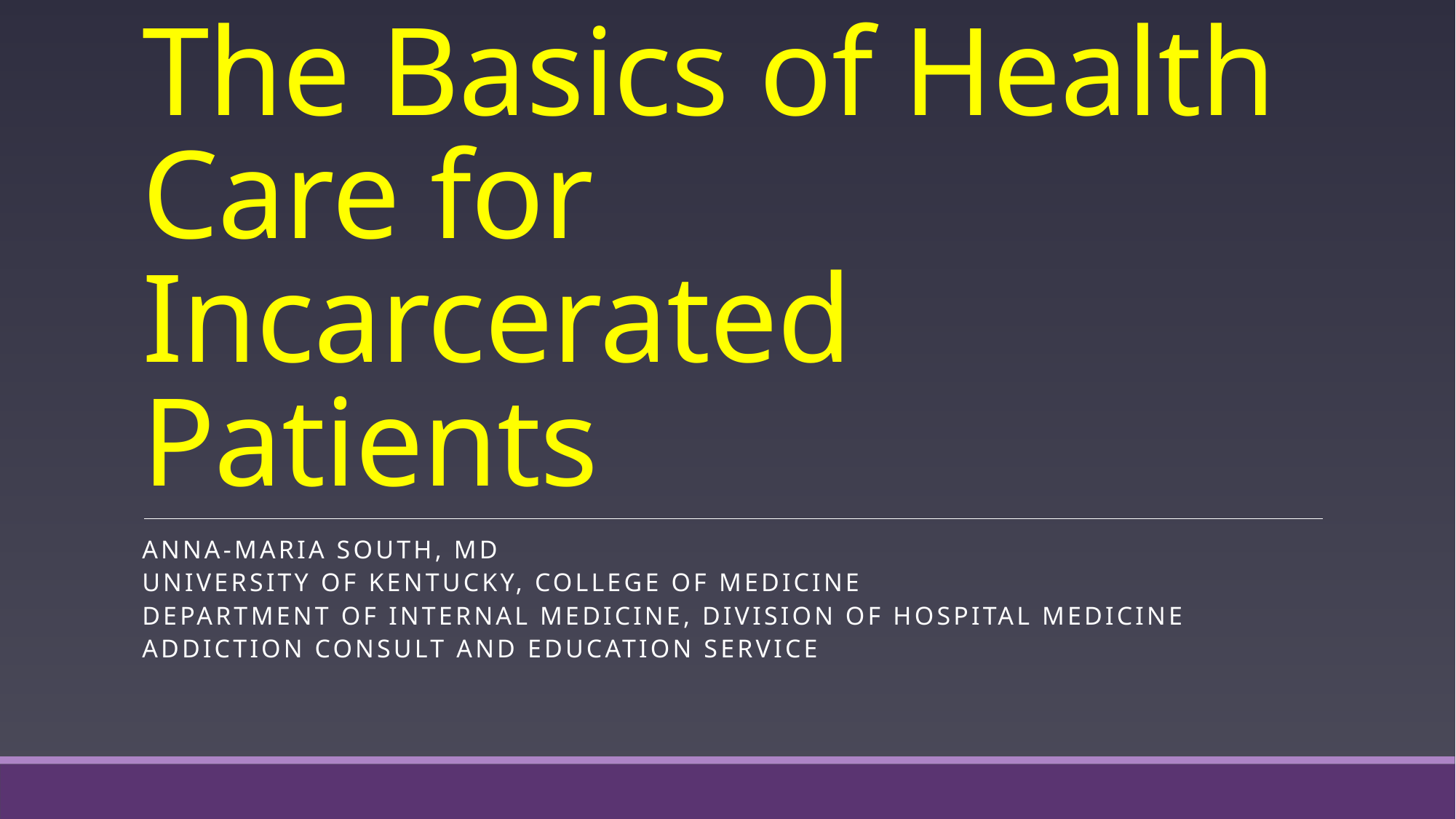

# The Basics of Health Care for Incarcerated Patients
Anna-Maria South, MD
University of Kentucky, College of Medicine
Department of Internal Medicine, Division of Hospital Medicine
Addiction Consult and Education Service

## Slide 3
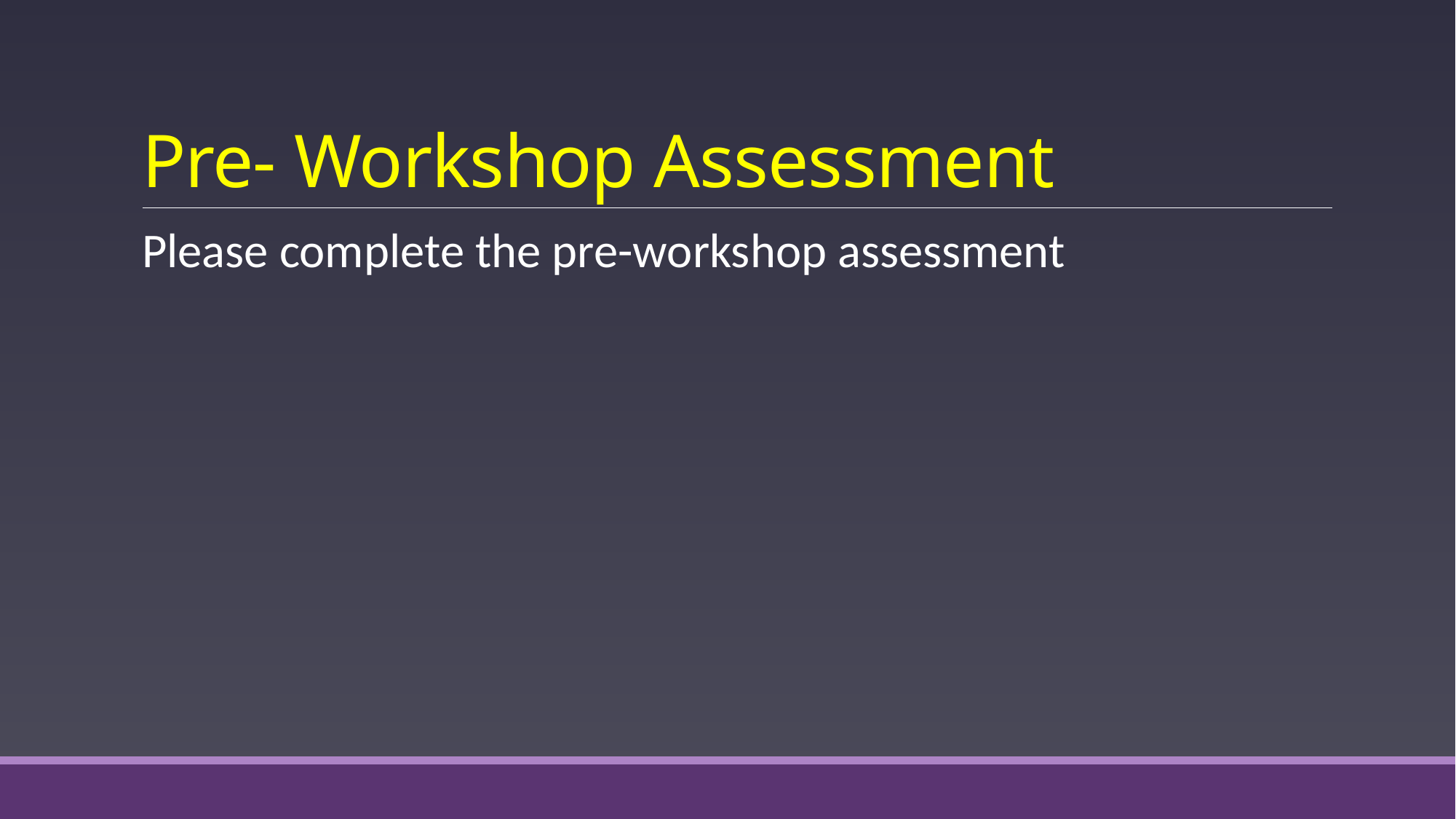

# Pre- Workshop Assessment
Please complete the pre-workshop assessment

## Slide 4
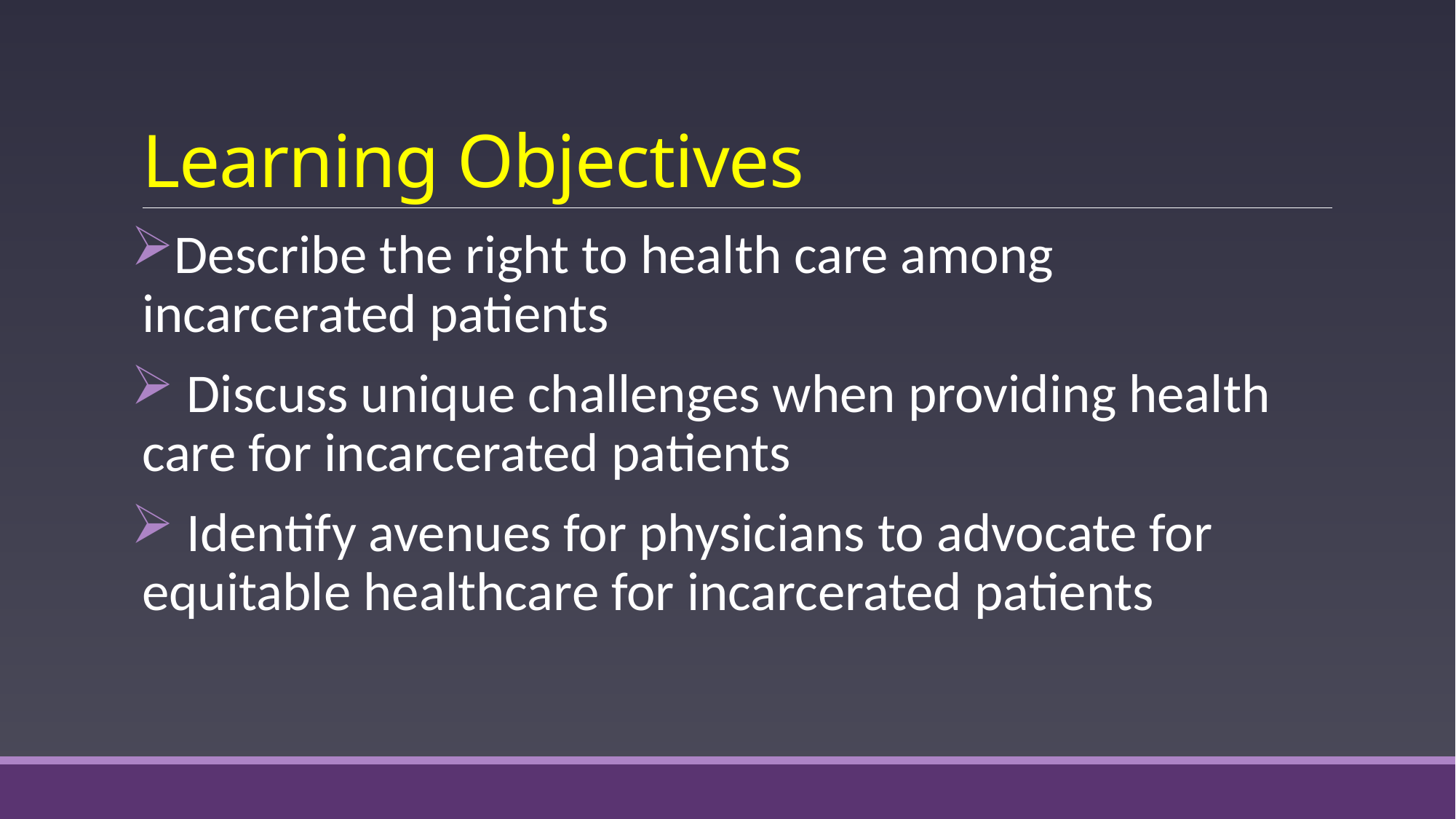

# Learning Objectives
Describe the right to health care among incarcerated patients
 Discuss unique challenges when providing health care for incarcerated patients
 Identify avenues for physicians to advocate for equitable healthcare for incarcerated patients

## Slide 5
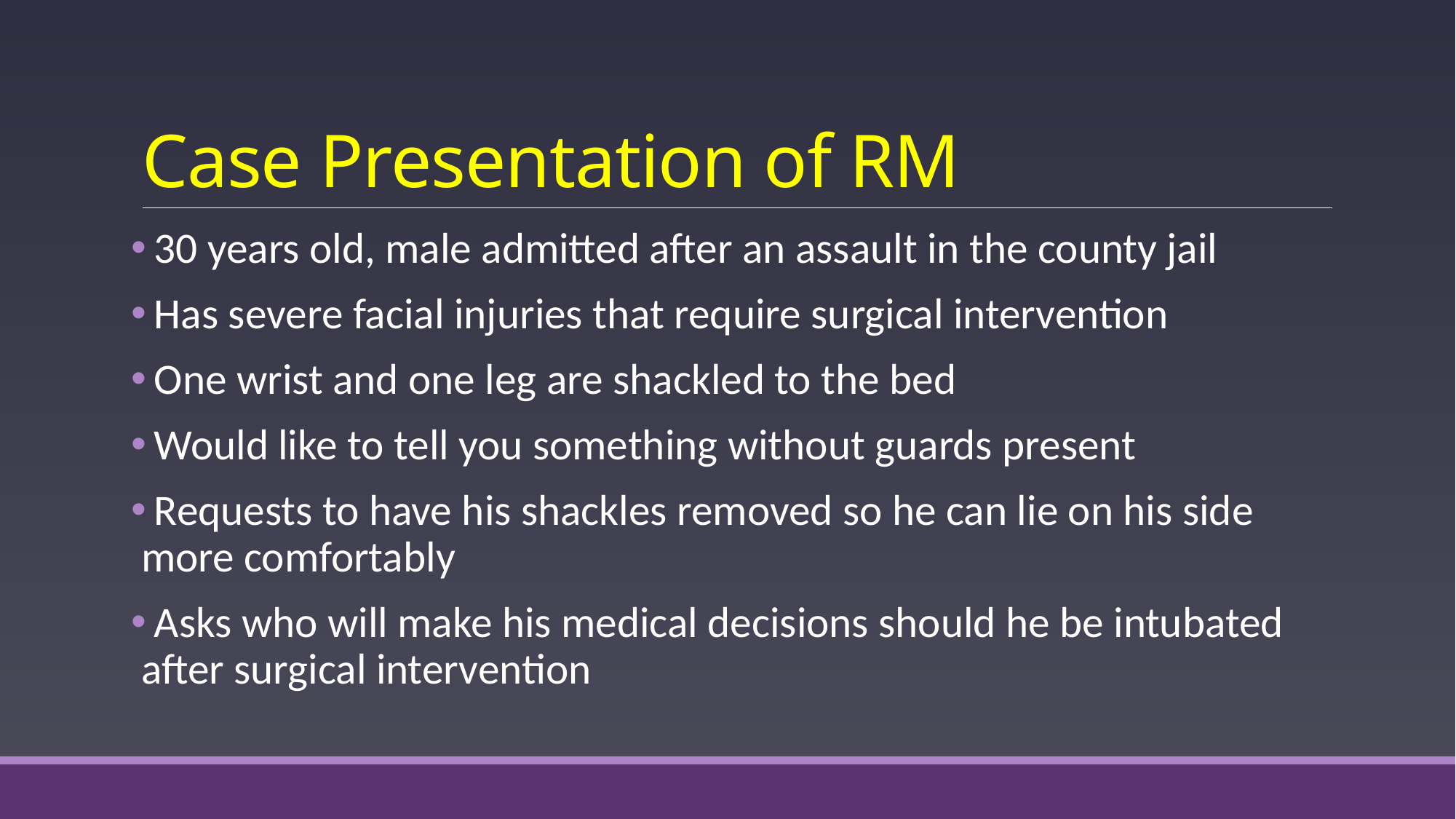

# Case Presentation of RM
 30 years old, male admitted after an assault in the county jail
 Has severe facial injuries that require surgical intervention
 One wrist and one leg are shackled to the bed
 Would like to tell you something without guards present
 Requests to have his shackles removed so he can lie on his side more comfortably
 Asks who will make his medical decisions should he be intubated after surgical intervention

## Slide 6
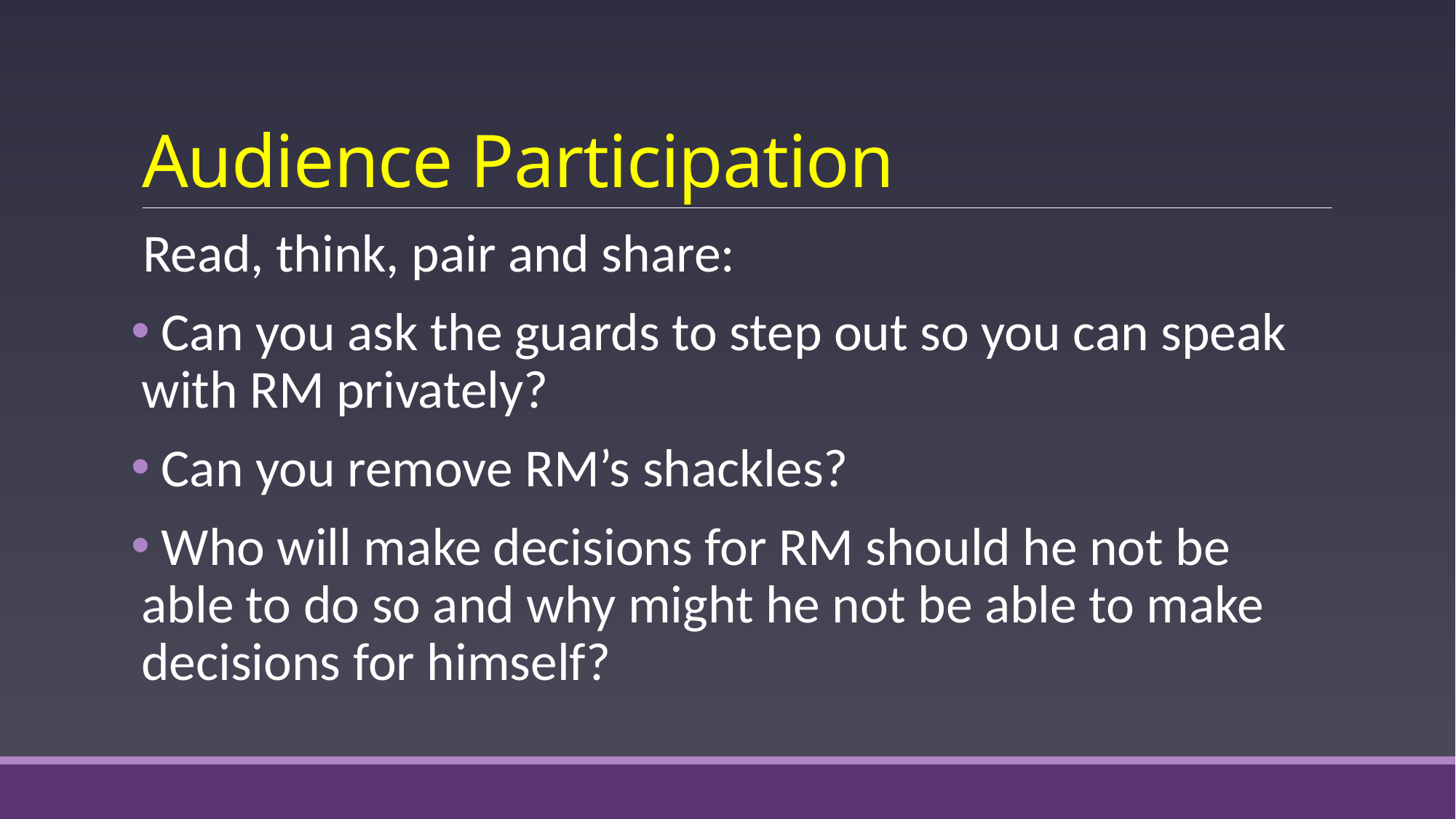

# Audience Participation
Read, think, pair and share:
 Can you ask the guards to step out so you can speak with RM privately?
 Can you remove RM’s shackles?
 Who will make decisions for RM should he not be able to do so and why might he not be able to make decisions for himself?

## Slide 7
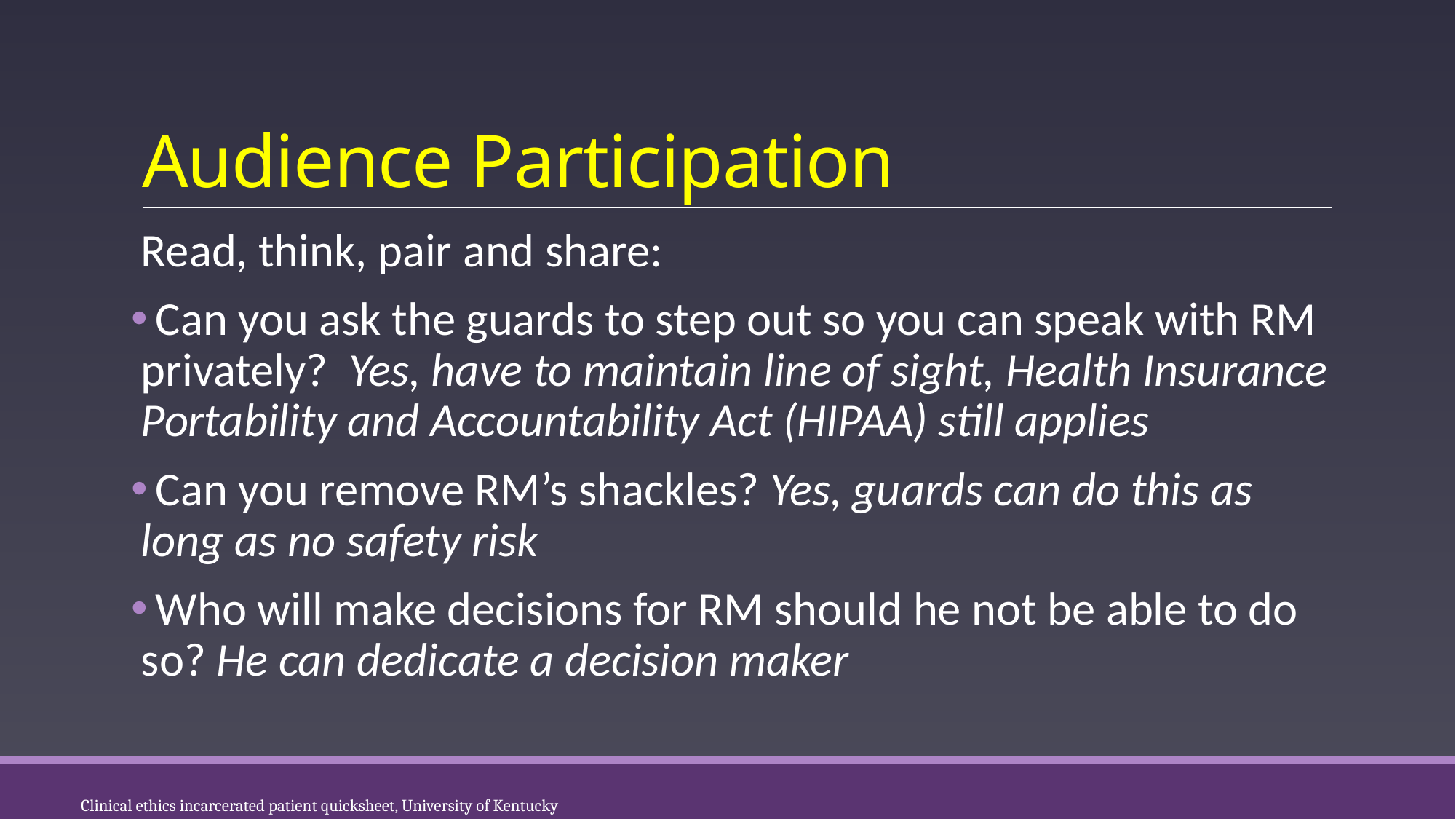

# Audience Participation
Read, think, pair and share:
 Can you ask the guards to step out so you can speak with RM privately?  Yes, have to maintain line of sight, Health Insurance Portability and Accountability Act (HIPAA) still applies
 Can you remove RM’s shackles? Yes, guards can do this as long as no safety risk
 Who will make decisions for RM should he not be able to do so? He can dedicate a decision maker
Clinical ethics incarcerated patient quicksheet, University of Kentucky

## Slide 8
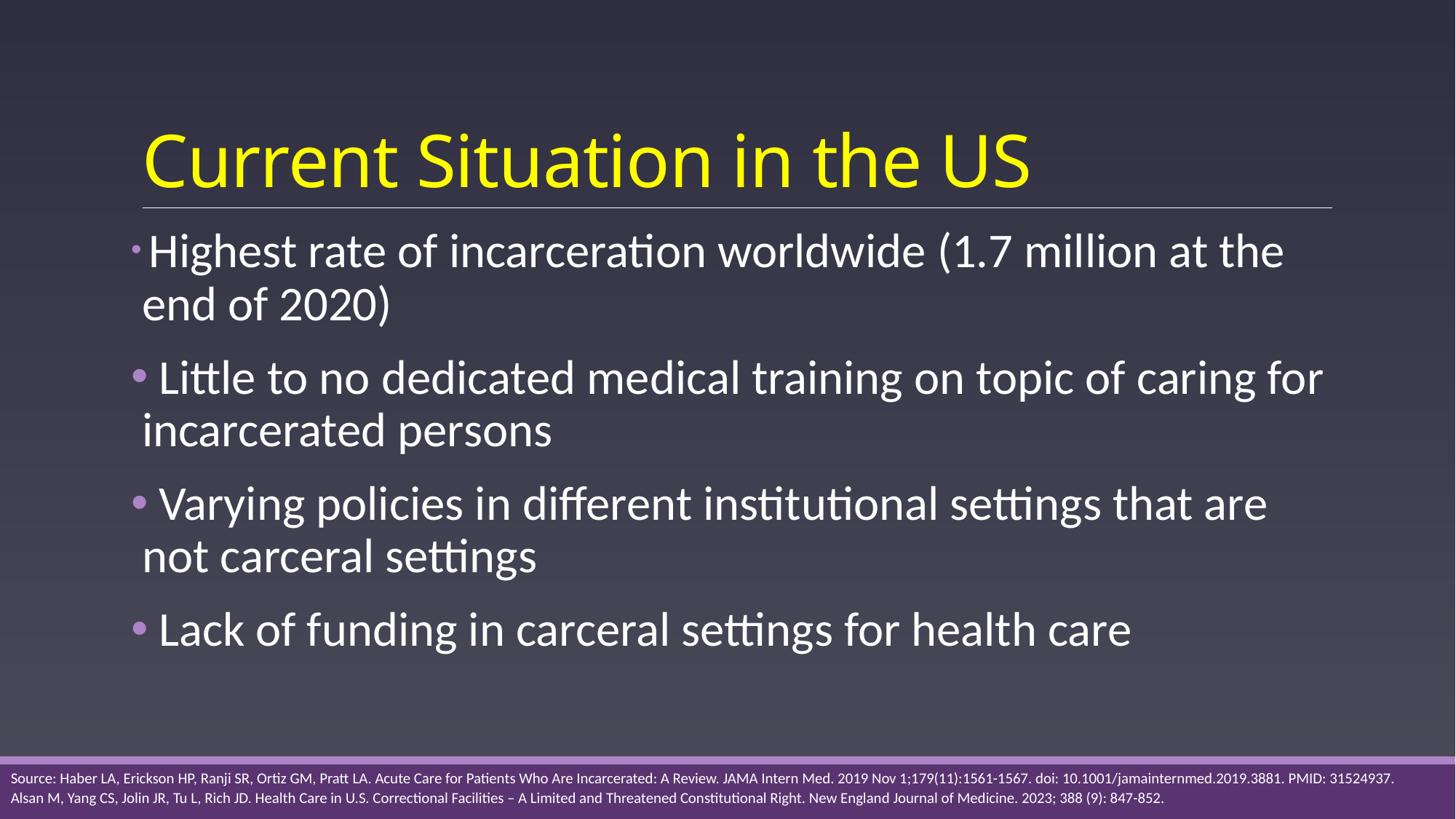

# Current Situation in the US
 Highest rate of incarceration worldwide (1.7 million at the end of 2020)
 Little to no dedicated medical training on topic of caring for incarcerated persons
 Varying policies in different institutional settings that are not carceral settings
 Lack of funding in carceral settings for health care
Source: Haber LA, Erickson HP, Ranji SR, Ortiz GM, Pratt LA. Acute Care for Patients Who Are Incarcerated: A Review. JAMA Intern Med. 2019 Nov 1;179(11):1561-1567. doi: 10.1001/jamainternmed.2019.3881. PMID: 31524937.
Alsan M, Yang CS, Jolin JR, Tu L, Rich JD. Health Care in U.S. Correctional Facilities – A Limited and Threatened Constitutional Right. New England Journal of Medicine. 2023; 388 (9): 847-852.

## Slide 9
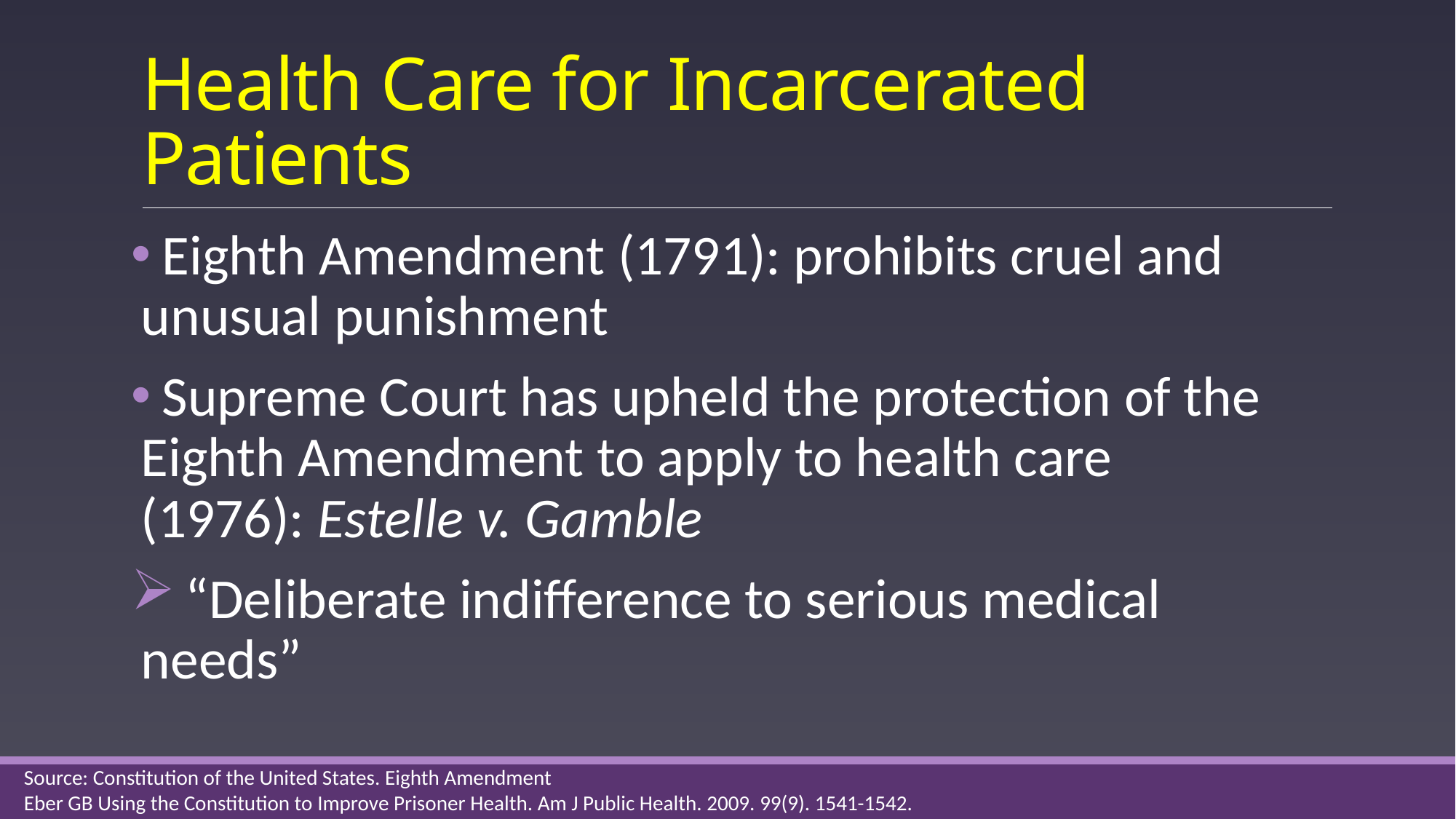

# Health Care for Incarcerated Patients
 Eighth Amendment (1791): prohibits cruel and unusual punishment
 Supreme Court has upheld the protection of the Eighth Amendment to apply to health care (1976): Estelle v. Gamble
 “Deliberate indifference to serious medical needs”
Source: Constitution of the United States. Eighth Amendment​
Eber GB Using the Constitution to Improve Prisoner Health. Am J Public Health. 2009. 99(9). 1541-1542.

## Slide 10
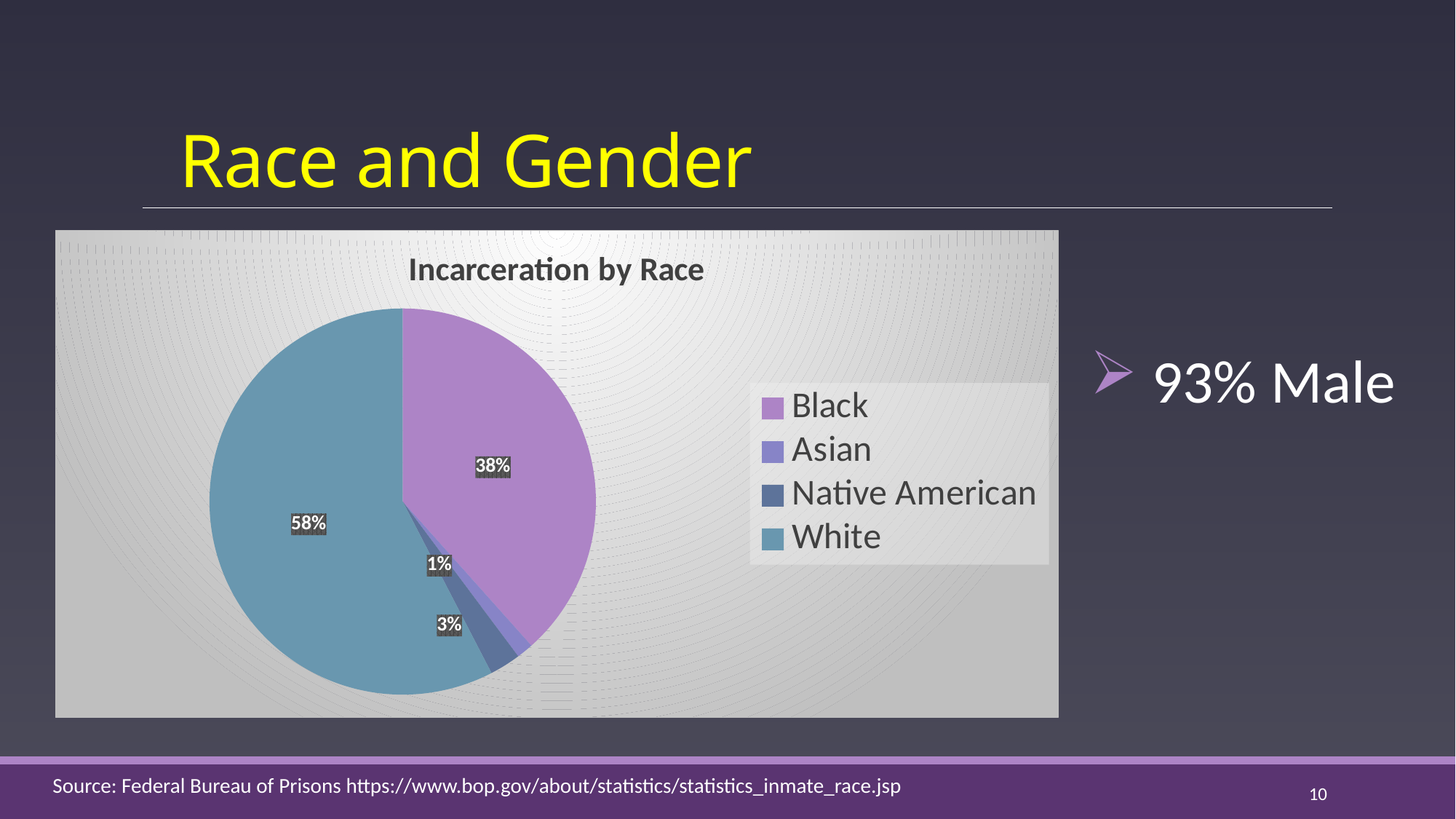

# Race and Gender
### Chart:
| Category | Incarceration by Race |
|---|---|
| Black | 38.4 |
| Asian | 1.4 |
| Native American | 2.6 |
| White | 57.6 | 93% Male
Source: Federal Bureau of Prisons https://www.bop.gov/about/statistics/statistics_inmate_race.jsp
10

## Slide 11
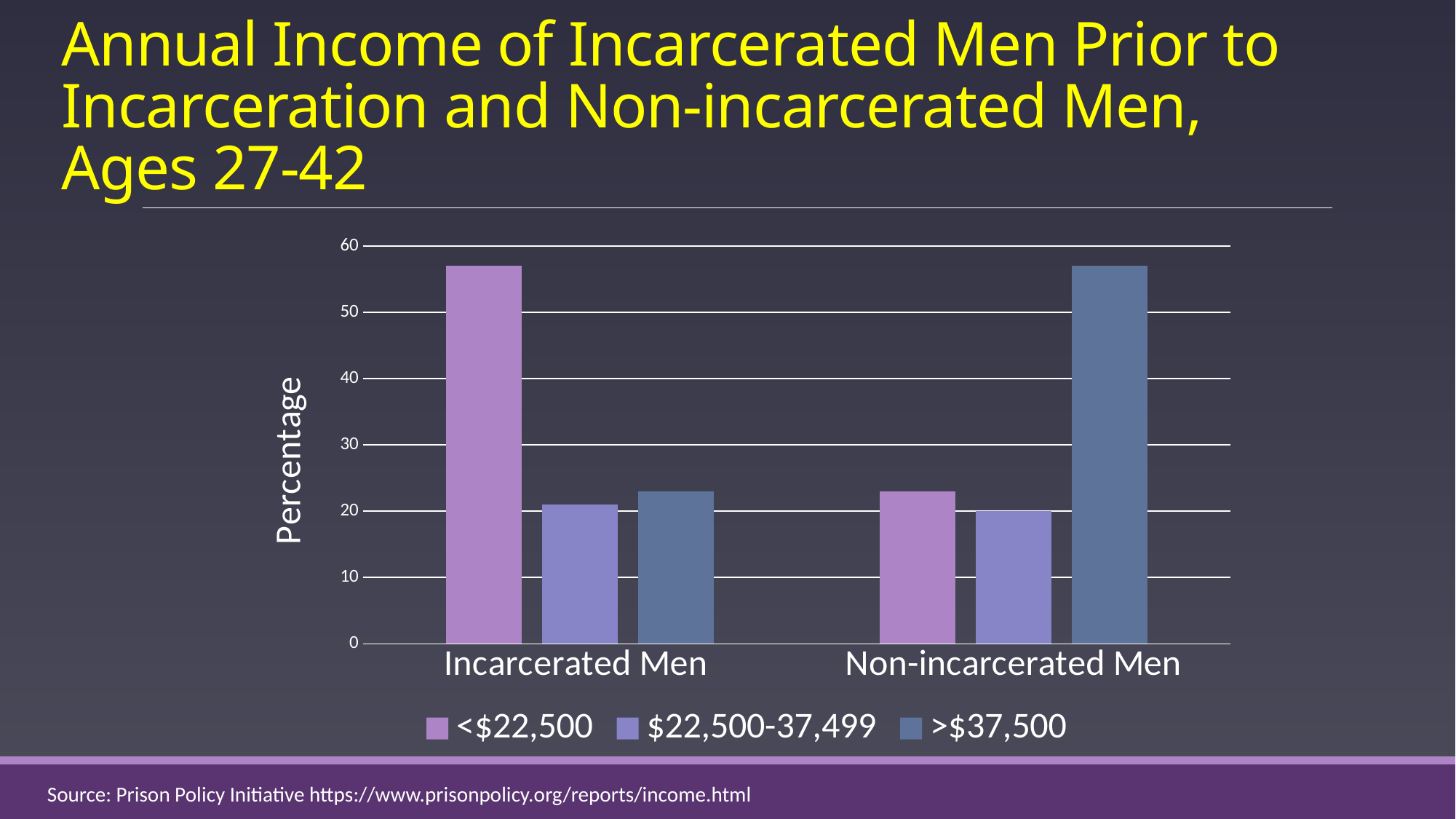

# Annual Income of Incarcerated Men Prior to Incarceration and Non-incarcerated Men, Ages 27-42
### Chart
| Category | <$22,500 | $22,500-37,499 | >$37,500 |
|---|---|---|---|
| Incarcerated Men | 57.0 | 21.0 | 23.0 |
| Non-incarcerated Men | 23.0 | 20.0 | 57.0 |Source: Prison Policy Initiative https://www.prisonpolicy.org/reports/income.html

## Slide 12
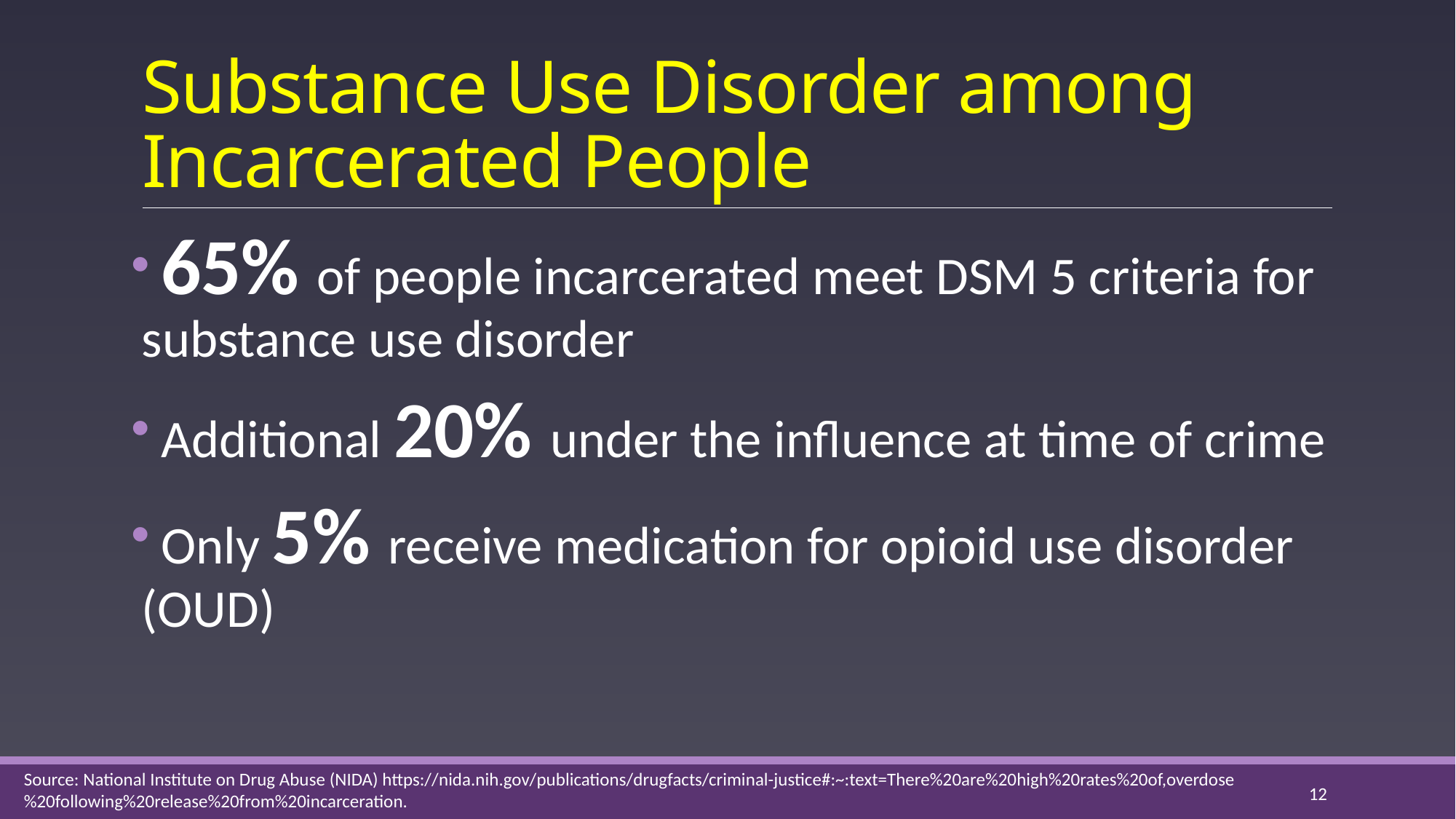

# Substance Use Disorder among  Incarcerated People
 65% of people incarcerated meet DSM 5 criteria for substance use disorder
 Additional 20% under the influence at time of crime
 Only 5% receive medication for opioid use disorder (OUD)
Source: National Institute on Drug Abuse (NIDA) https://nida.nih.gov/publications/drugfacts/criminal-justice#:~:text=There%20are%20high%20rates%20of,overdose%20following%20release%20from%20incarceration.
12

## Slide 13
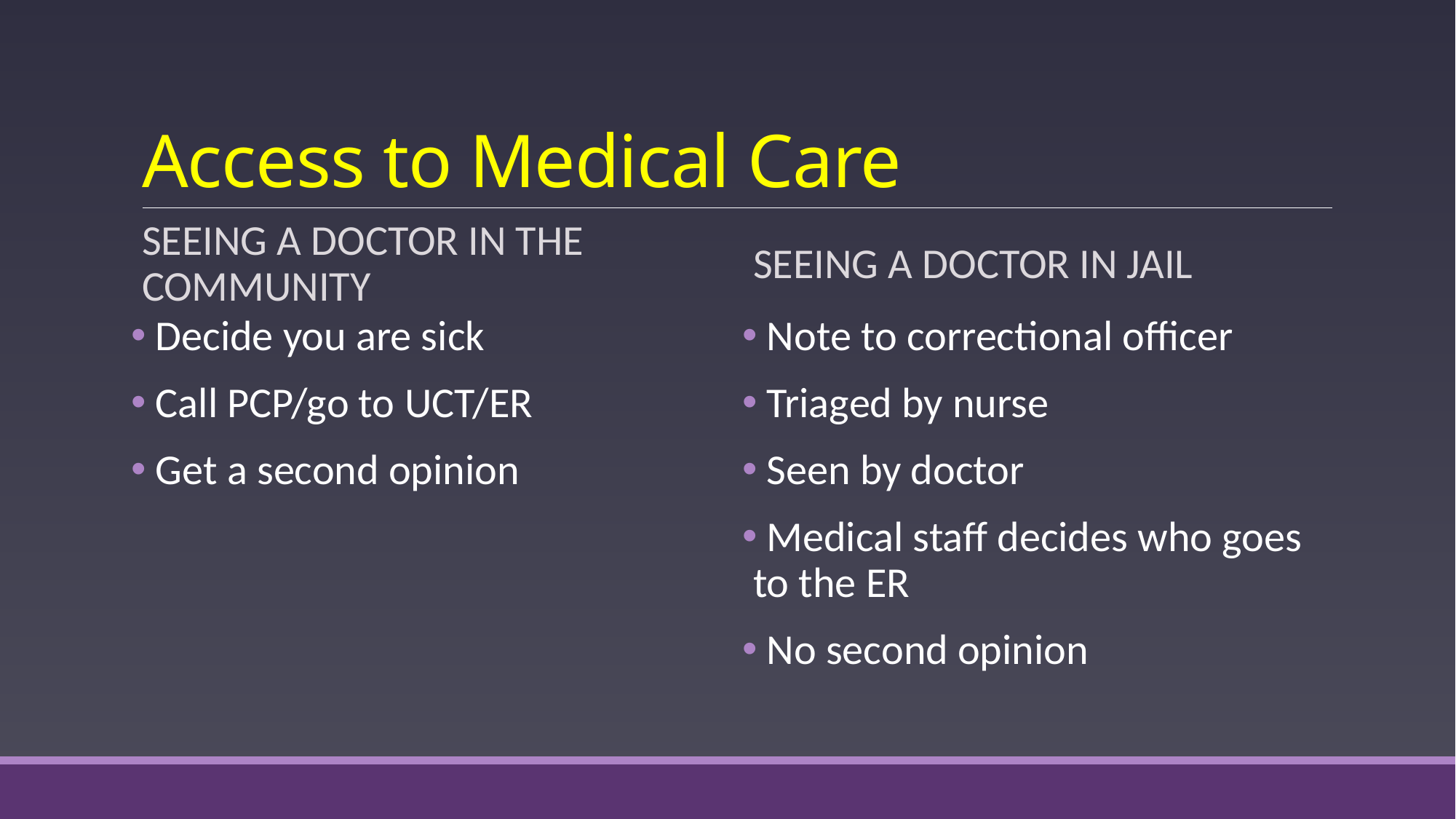

# Access to Medical Care
Seeing a doctor in the community
Seeing a doctor in Jail
 Decide you are sick
 Call PCP/go to UCT/ER
 Get a second opinion
 Note to correctional officer
 Triaged by nurse
 Seen by doctor
 Medical staff decides who goes to the ER
 No second opinion

## Slide 14
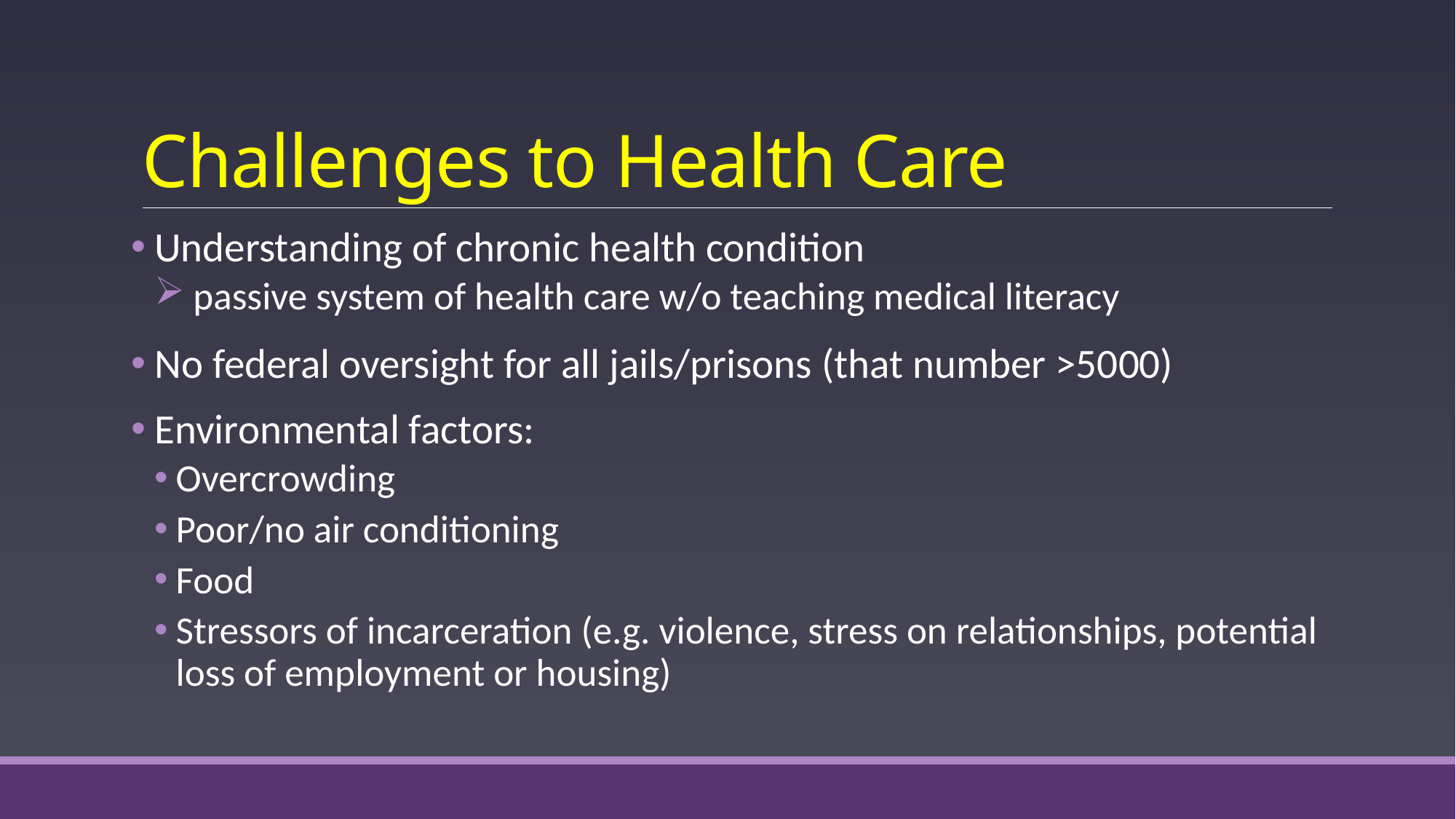

# Challenges to Health Care
 Understanding of chronic health condition
 passive system of health care w/o teaching medical literacy
 No federal oversight for all jails/prisons (that number >5000)
 Environmental factors:
Overcrowding
Poor/no air conditioning
Food
Stressors of incarceration (e.g. violence, stress on relationships, potential loss of employment or housing)

## Slide 15
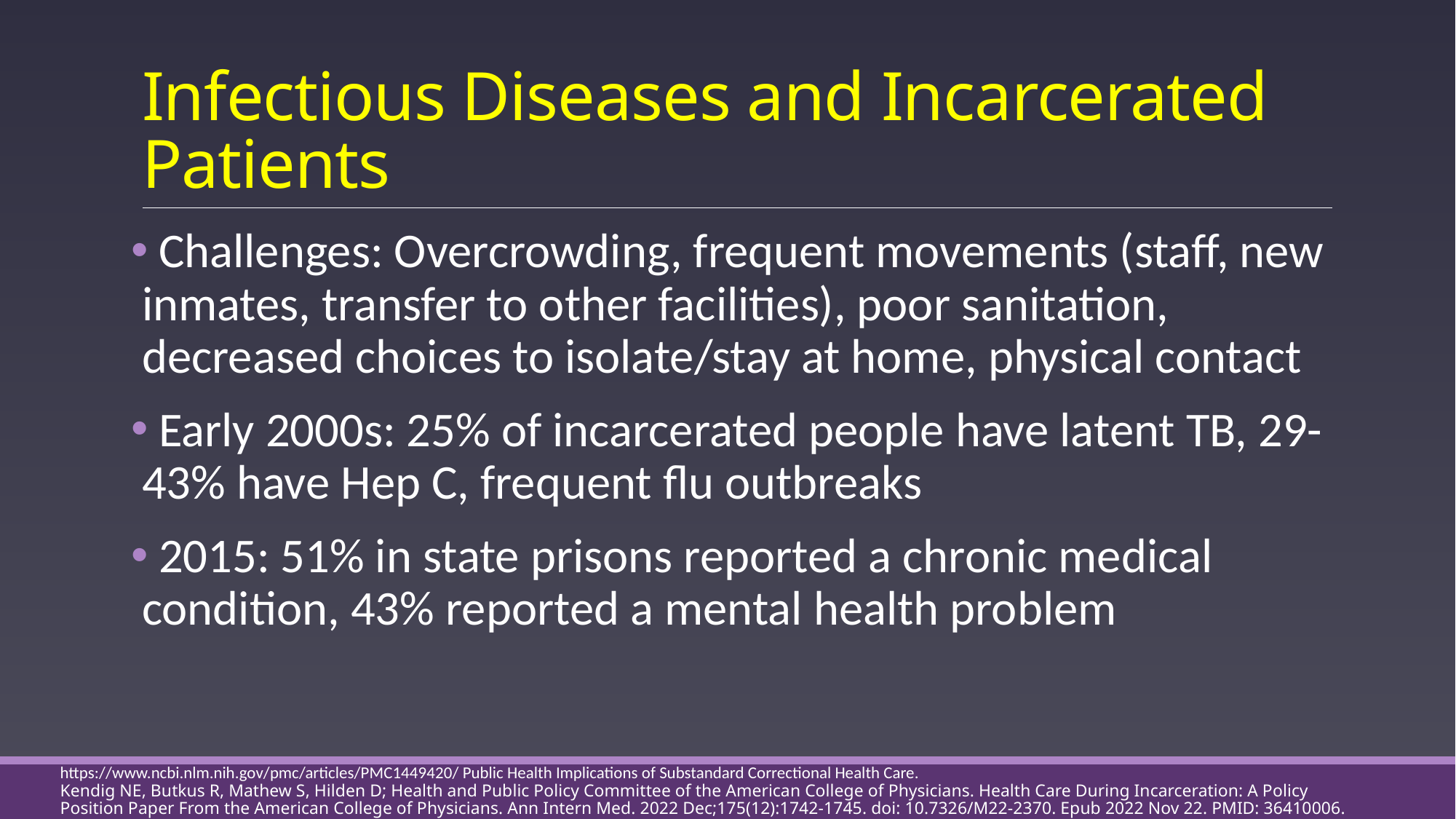

# Infectious Diseases and Incarcerated Patients
 Challenges: Overcrowding, frequent movements (staff, new inmates, transfer to other facilities), poor sanitation, decreased choices to isolate/stay at home, physical contact
 Early 2000s: 25% of incarcerated people have latent TB, 29-43% have Hep C, frequent flu outbreaks
 2015: 51% in state prisons reported a chronic medical condition, 43% reported a mental health problem
https://www.ncbi.nlm.nih.gov/pmc/articles/PMC1449420/ Public Health Implications of Substandard Correctional Health Care.
Kendig NE, Butkus R, Mathew S, Hilden D; Health and Public Policy Committee of the American College of Physicians. Health Care During Incarceration: A Policy Position Paper From the American College of Physicians. Ann Intern Med. 2022 Dec;175(12):1742-1745. doi: 10.7326/M22-2370. Epub 2022 Nov 22. PMID: 36410006.

## Slide 16
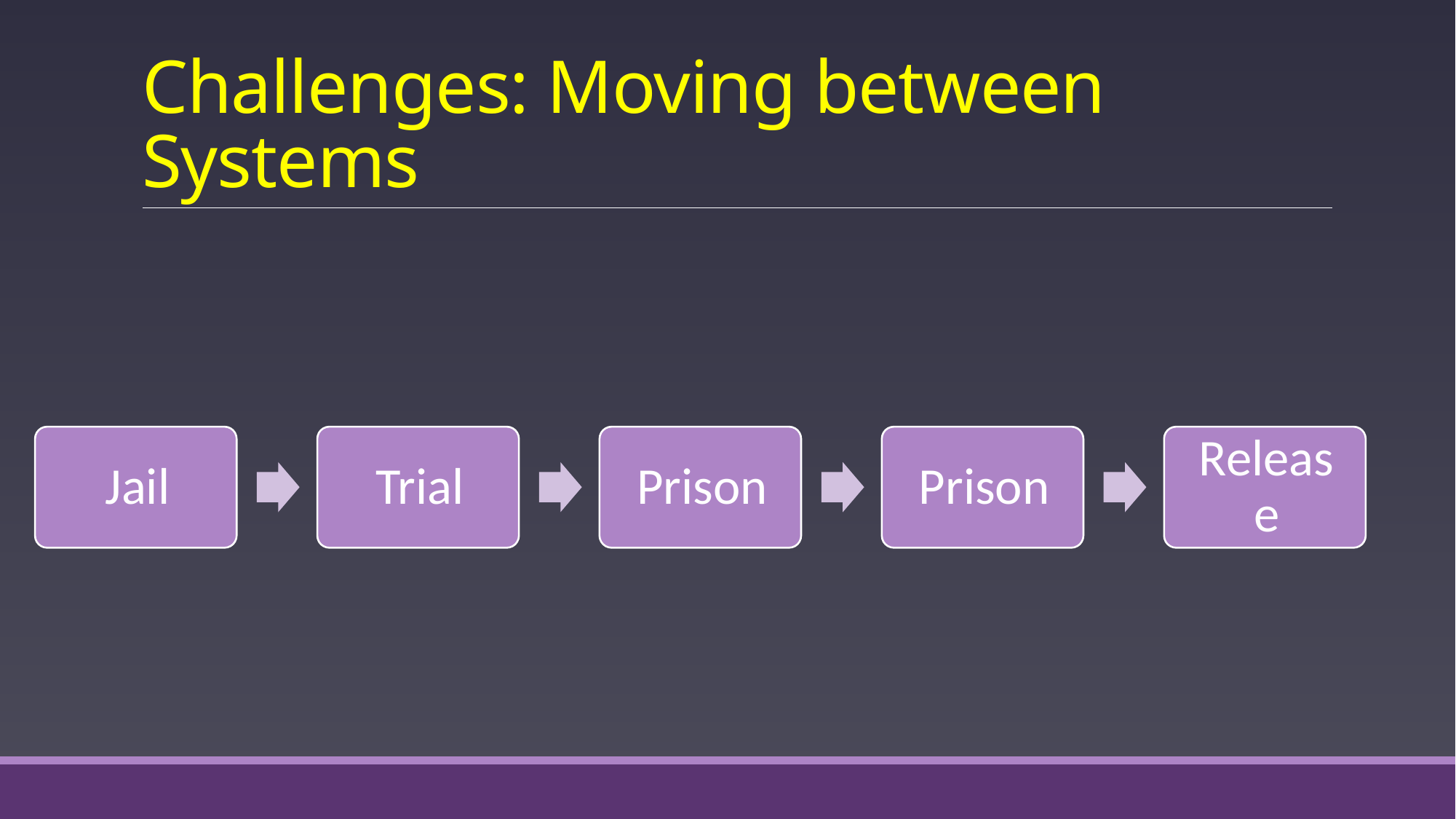

# Challenges: Moving between Systems

## Slide 17
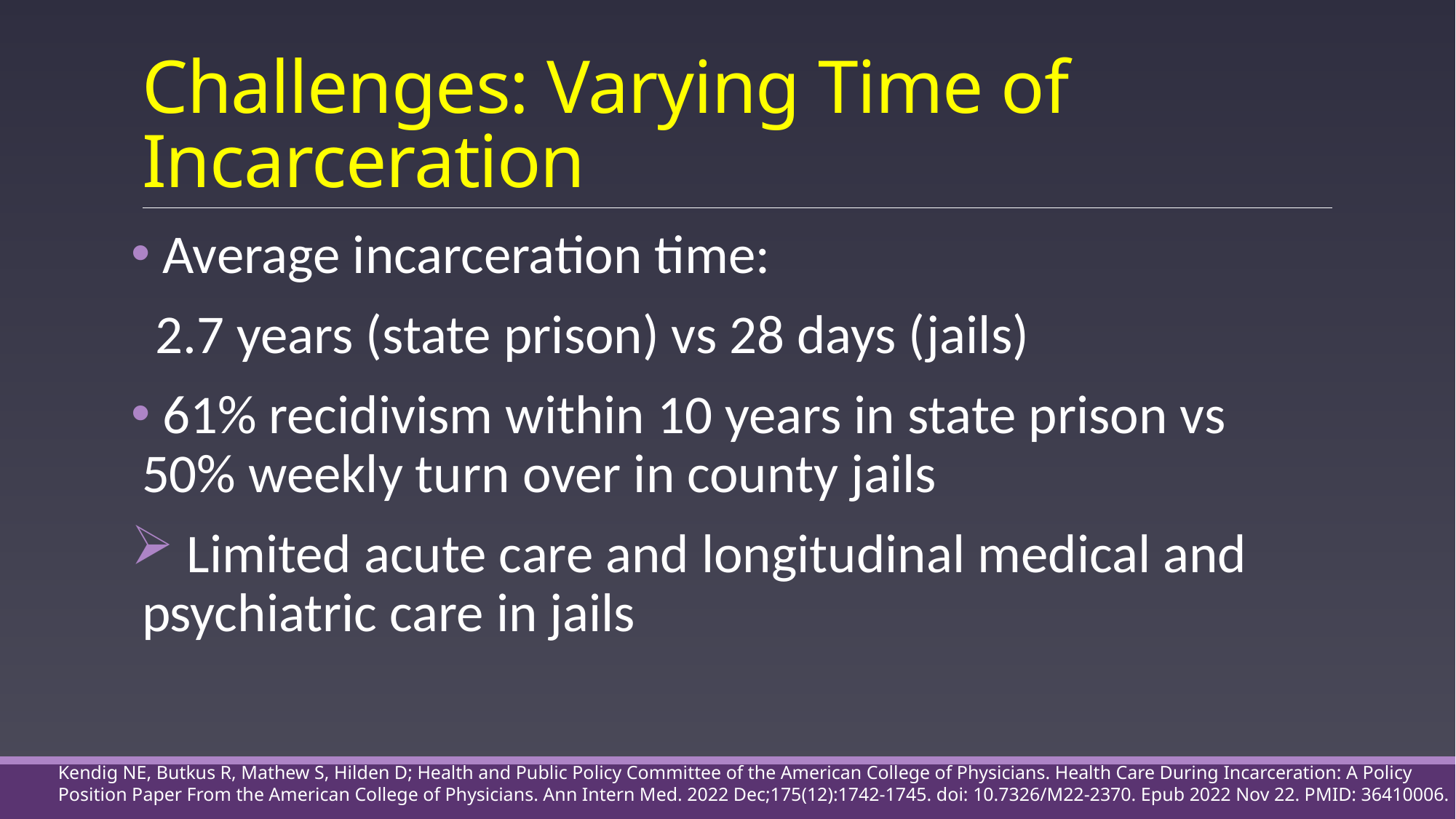

# Challenges: Varying Time of Incarceration
 Average incarceration time:
 2.7 years (state prison) vs 28 days (jails)
 61% recidivism within 10 years in state prison vs 50% weekly turn over in county jails
 Limited acute care and longitudinal medical and psychiatric care in jails
Kendig NE, Butkus R, Mathew S, Hilden D; Health and Public Policy Committee of the American College of Physicians. Health Care During Incarceration: A Policy Position Paper From the American College of Physicians. Ann Intern Med. 2022 Dec;175(12):1742-1745. doi: 10.7326/M22-2370. Epub 2022 Nov 22. PMID: 36410006.

## Slide 18
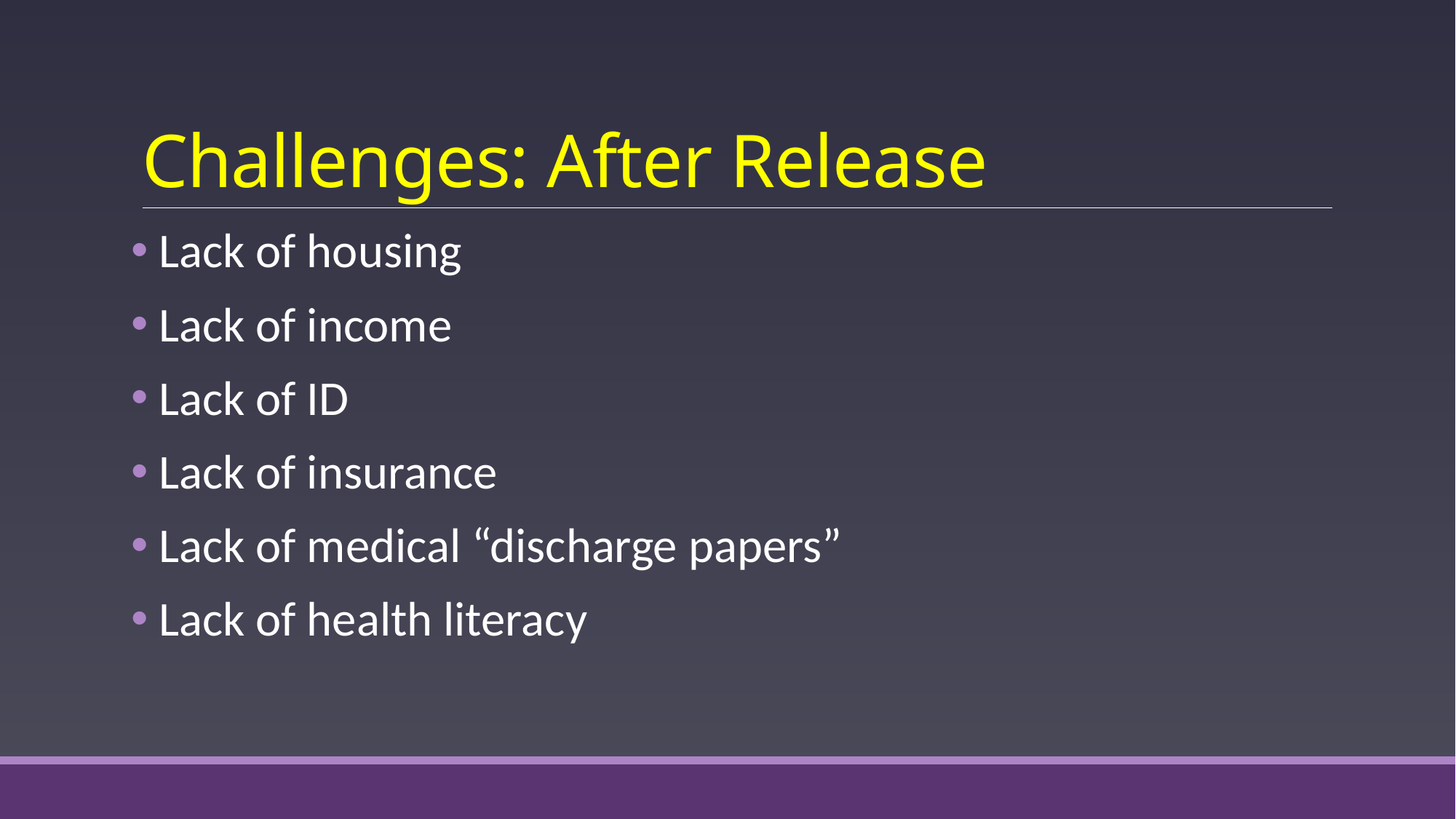

# Challenges: After Release
 Lack of housing
 Lack of income
 Lack of ID
 Lack of insurance
 Lack of medical “discharge papers”
 Lack of health literacy

## Slide 19
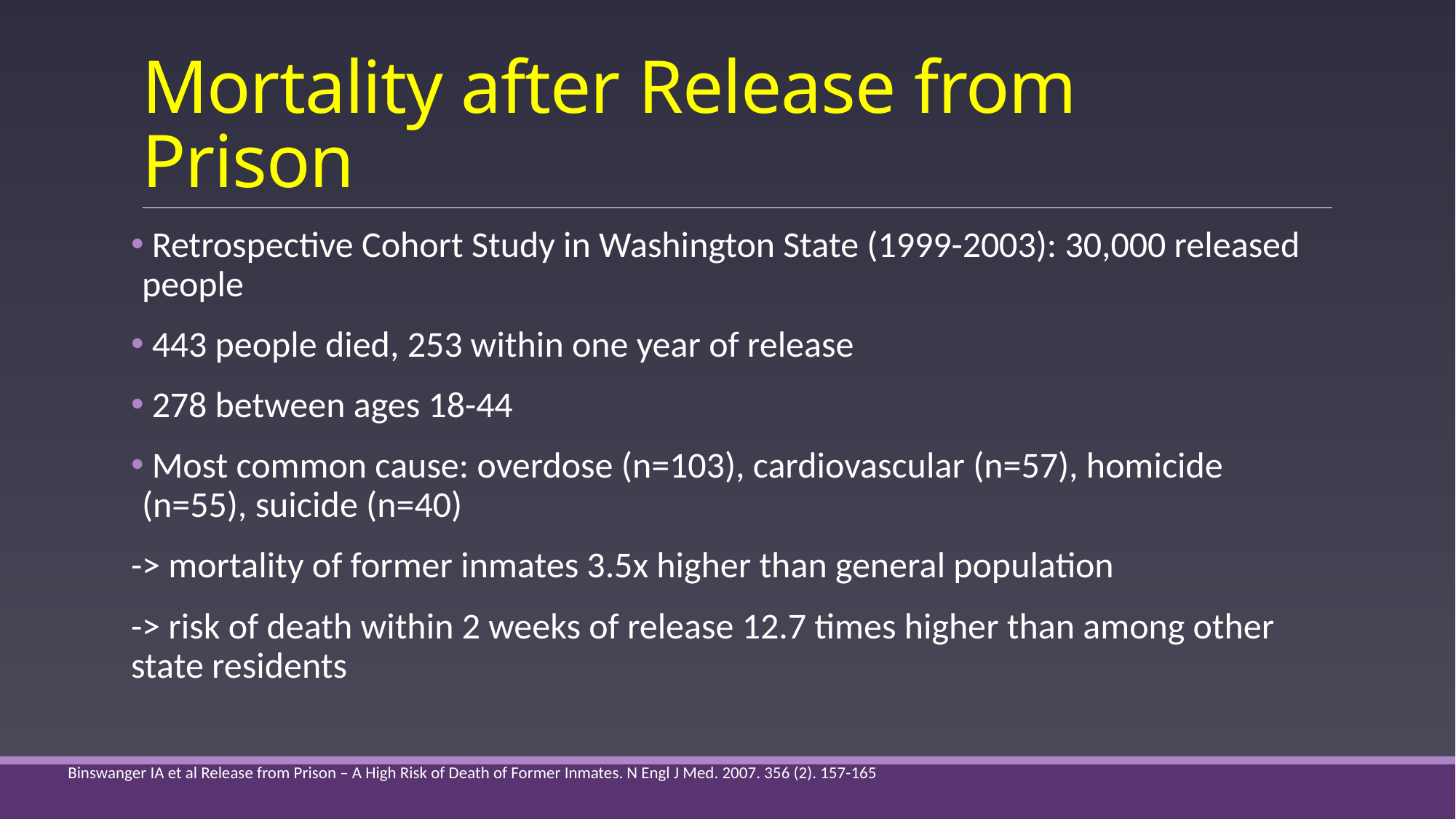

# Mortality after Release from Prison
 Retrospective Cohort Study in Washington State (1999-2003): 30,000 released people
 443 people died, 253 within one year of release
 278 between ages 18-44
 Most common cause: overdose (n=103), cardiovascular (n=57), homicide (n=55), suicide (n=40)
-> mortality of former inmates 3.5x higher than general population
-> risk of death within 2 weeks of release 12.7 times higher than among other state residents
Binswanger IA et al Release from Prison – A High Risk of Death of Former Inmates. N Engl J Med. 2007. 356 (2). 157-165

## Slide 20
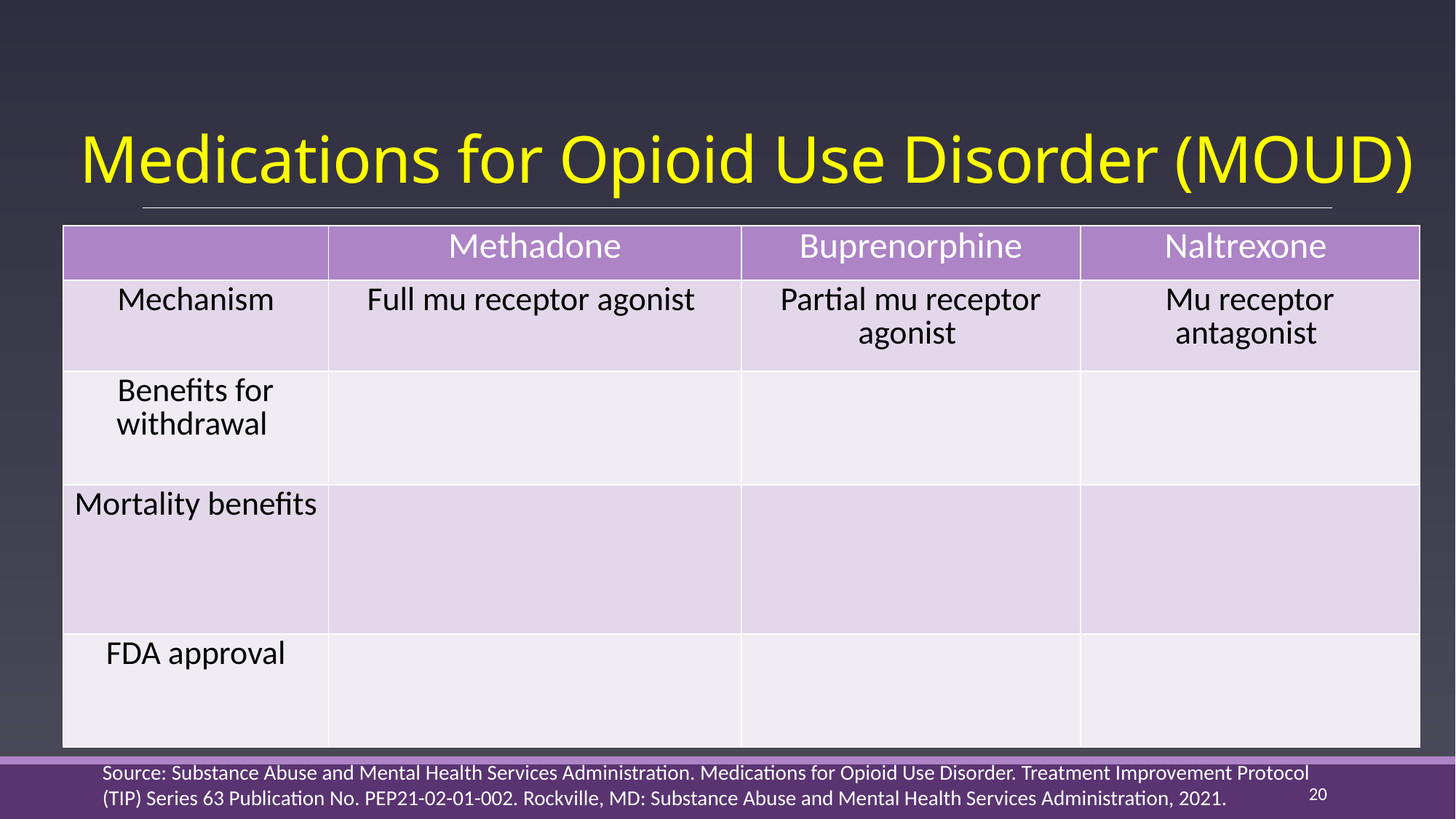

# Medications for Opioid Use Disorder (MOUD)
| | Methadone | Buprenorphine | Naltrexone |
| --- | --- | --- | --- |
| Mechanism | Full mu receptor agonist | Partial mu receptor agonist | Mu receptor antagonist |
| Benefits for withdrawal | | | |
| Mortality benefits | | | |
| FDA approval | | | |
Source: Substance Abuse and Mental Health Services Administration. Medications for Opioid Use Disorder. Treatment Improvement Protocol (TIP) Series 63 Publication No. PEP21-02-01-002. Rockville, MD: Substance Abuse and Mental Health Services Administration, 2021.
20

## Slide 21
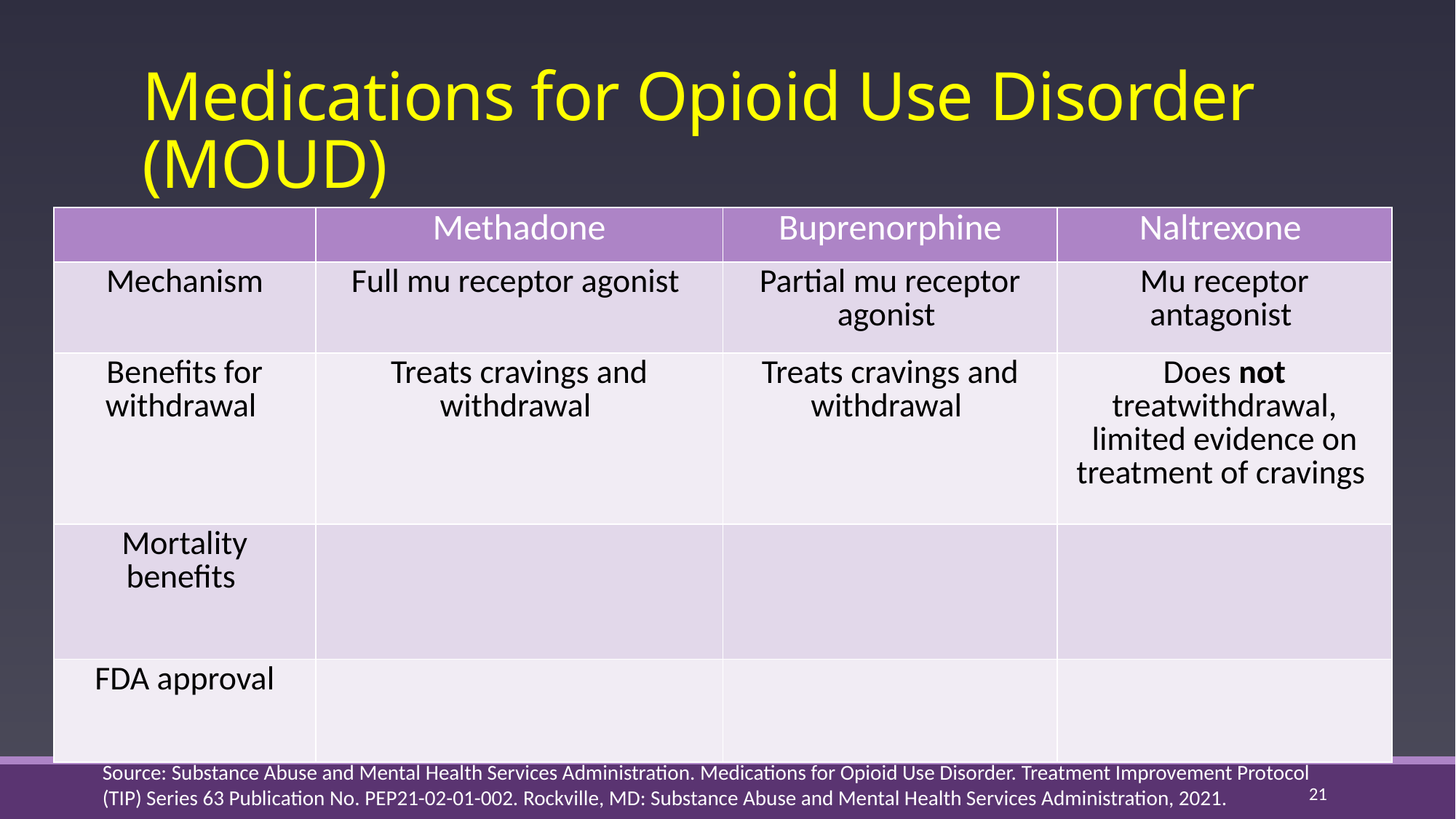

# Medications for Opioid Use Disorder (MOUD)
| | Methadone | Buprenorphine | Naltrexone |
| --- | --- | --- | --- |
| Mechanism | Full mu receptor agonist | Partial mu receptor agonist | Mu receptor antagonist |
| Benefits for withdrawal | Treats cravings and withdrawal | Treats cravings and withdrawal | Does not treatwithdrawal, limited evidence on treatment of cravings |
| Mortality benefits | | | |
| FDA approval | | | |
Source: Substance Abuse and Mental Health Services Administration. Medications for Opioid Use Disorder. Treatment Improvement Protocol (TIP) Series 63 Publication No. PEP21-02-01-002. Rockville, MD: Substance Abuse and Mental Health Services Administration, 2021.
21

## Slide 22
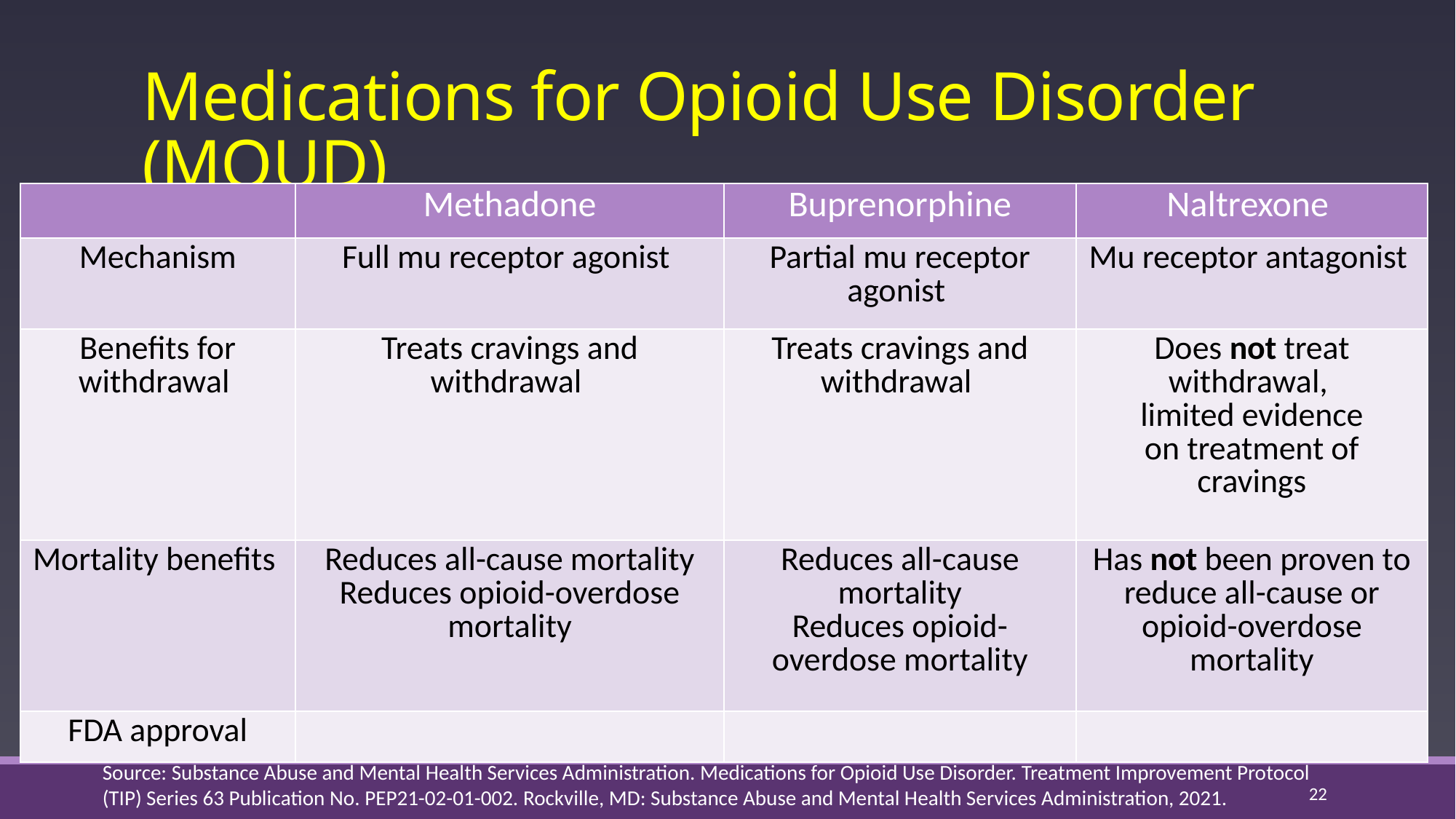

# Medications for Opioid Use Disorder (MOUD)
| | Methadone | Buprenorphine | Naltrexone |
| --- | --- | --- | --- |
| Mechanism | Full mu receptor agonist | Partial mu receptor agonist | Mu receptor antagonist |
| Benefits for withdrawal | Treats cravings and withdrawal | Treats cravings and withdrawal | Does not treat withdrawal,  limited evidence on treatment of cravings |
| Mortality benefits | Reduces all-cause mortality Reduces opioid-overdose mortality | Reduces all-cause mortality Reduces opioid-overdose mortality | Has not been proven to reduce all-cause or opioid-overdose mortality |
| FDA approval | | | |
Source: Substance Abuse and Mental Health Services Administration. Medications for Opioid Use Disorder. Treatment Improvement Protocol (TIP) Series 63 Publication No. PEP21-02-01-002. Rockville, MD: Substance Abuse and Mental Health Services Administration, 2021.
22

## Slide 23
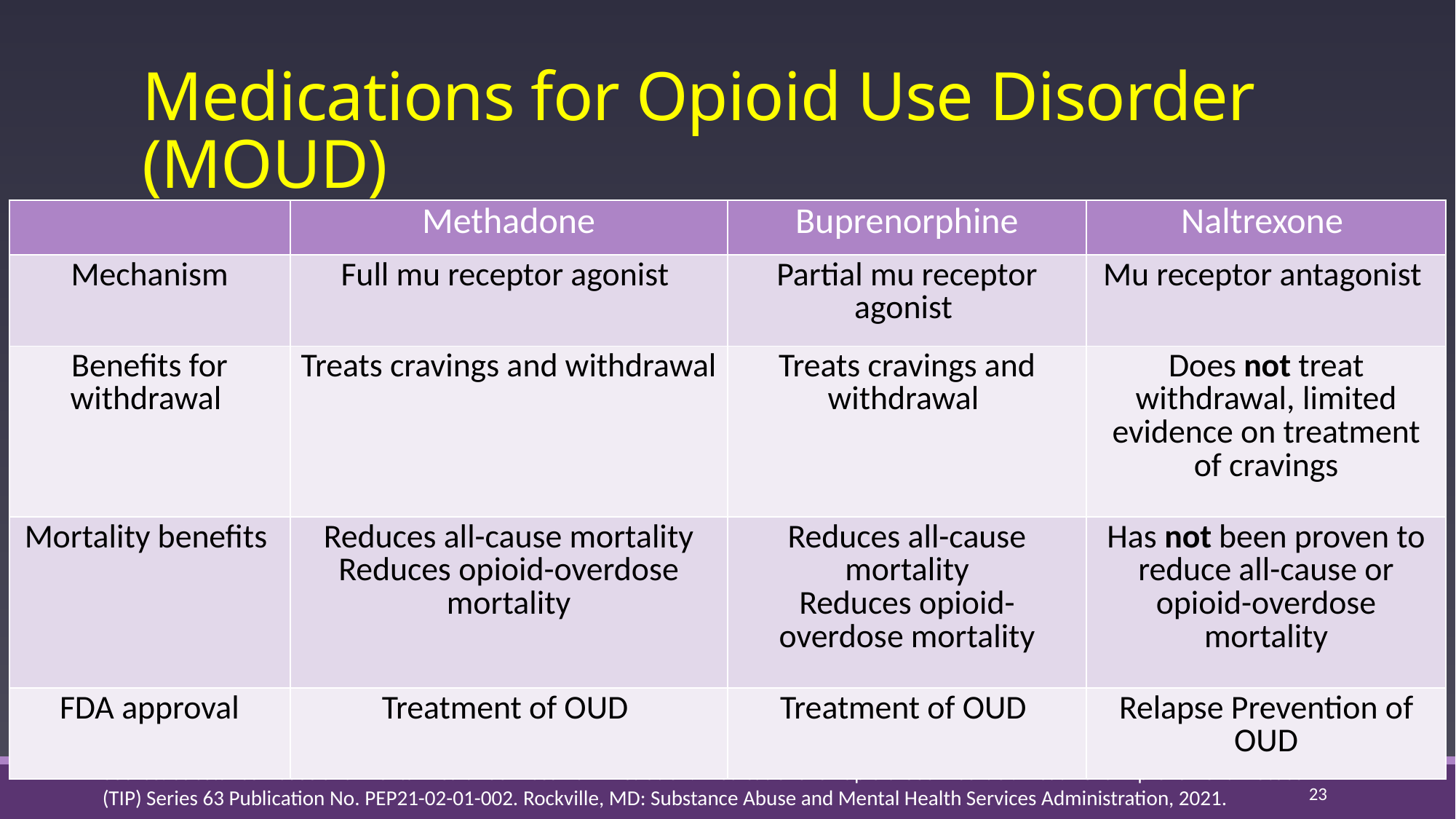

# Medications for Opioid Use Disorder (MOUD)
| | Methadone | Buprenorphine | Naltrexone |
| --- | --- | --- | --- |
| Mechanism | Full mu receptor agonist | Partial mu receptor agonist | Mu receptor antagonist |
| Benefits for withdrawal | Treats cravings and withdrawal | Treats cravings and withdrawal | Does not treat withdrawal, limited evidence on treatment of cravings |
| Mortality benefits | Reduces all-cause mortality Reduces opioid-overdose mortality | Reduces all-cause mortality Reduces opioid-overdose mortality | Has not been proven to reduce all-cause or opioid-overdose mortality |
| FDA approval | Treatment of OUD | Treatment of OUD | Relapse Prevention of OUD |
Source: Substance Abuse and Mental Health Services Administration. Medications for Opioid Use Disorder. Treatment Improvement Protocol (TIP) Series 63 Publication No. PEP21-02-01-002. Rockville, MD: Substance Abuse and Mental Health Services Administration, 2021.
23

## Slide 24
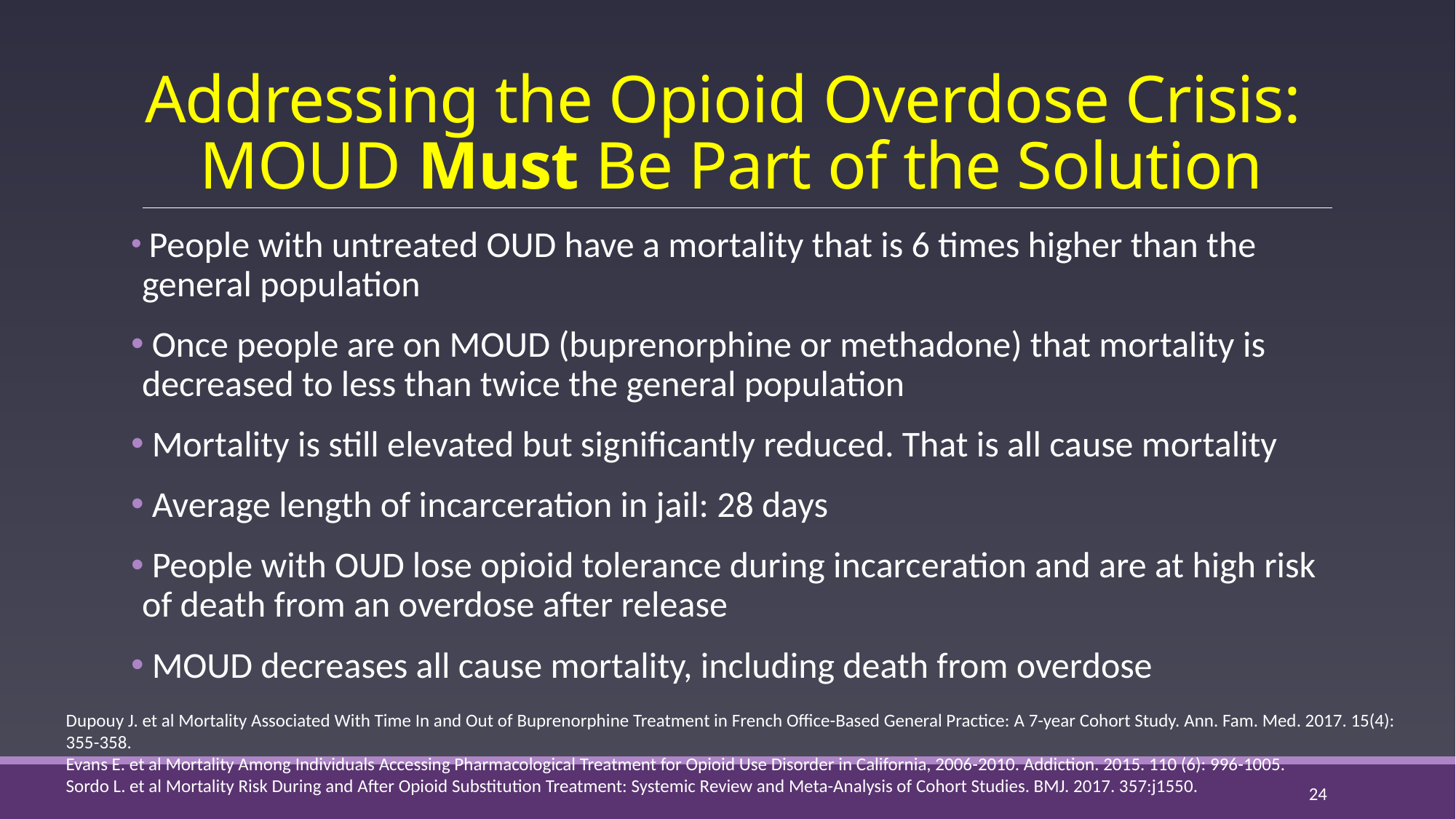

# Addressing the Opioid Overdose Crisis: MOUD Must Be Part of the Solution
 People with untreated OUD have a mortality that is 6 times higher than the general population
 Once people are on MOUD (buprenorphine or methadone) that mortality is decreased to less than twice the general population
 Mortality is still elevated but significantly reduced. That is all cause mortality
 Average length of incarceration in jail: 28 days
 People with OUD lose opioid tolerance during incarceration and are at high risk of death from an overdose after release
 MOUD decreases all cause mortality, including death from overdose
Dupouy J. et al Mortality Associated With Time In and Out of Buprenorphine Treatment in French Office-Based General Practice: A 7-year Cohort Study. Ann. Fam. Med. 2017. 15(4): 355-358.
Evans E. et al Mortality Among Individuals Accessing Pharmacological Treatment for Opioid Use Disorder in California, 2006-2010. Addiction. 2015. 110 (6): 996-1005.
Sordo L. et al Mortality Risk During and After Opioid Substitution Treatment: Systemic Review and Meta-Analysis of Cohort Studies. BMJ. 2017. 357:j1550.
24

## Slide 25
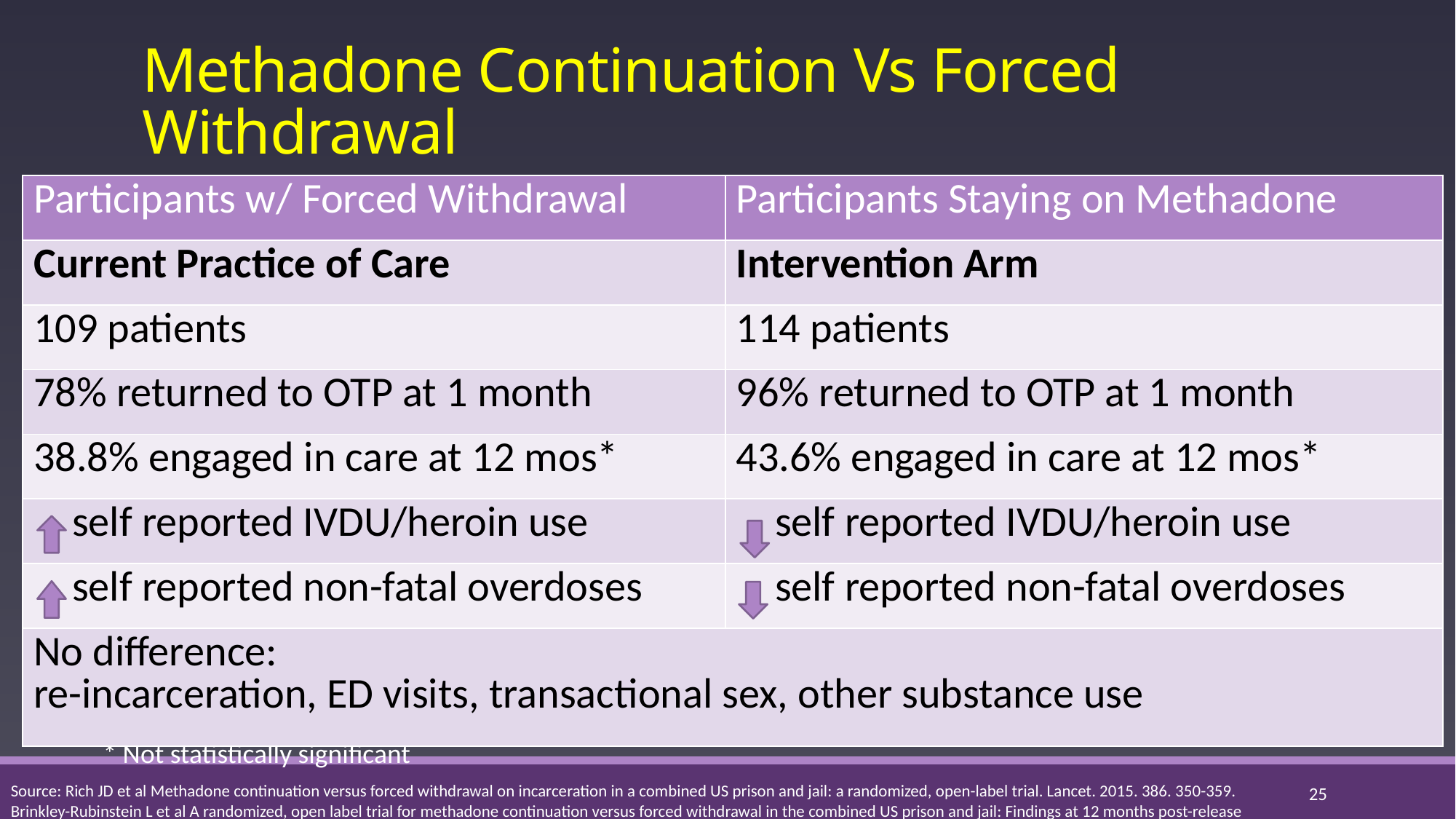

# Methadone Continuation Vs Forced Withdrawal
| Participants w/ Forced Withdrawal | Participants Staying on Methadone |
| --- | --- |
| Current Practice of Care | Intervention Arm |
| 109 patients | 114 patients |
| 78% returned to OTP at 1 month | 96% returned to OTP at 1 month |
| 38.8% engaged in care at 12 mos\* | 43.6% engaged in care at 12 mos\* |
| self reported IVDU/heroin use | self reported IVDU/heroin use |
| self reported non-fatal overdoses | self reported non-fatal overdoses |
| No difference: re-incarceration, ED visits, transactional sex, other substance use | |
* Not statistically significant
25
Source: Rich JD et al Methadone continuation versus forced withdrawal on incarceration in a combined US prison and jail: a randomized, open-label trial. Lancet. 2015. 386. 350-359.
Brinkley-Rubinstein L et al A randomized, open label trial for methadone continuation versus forced withdrawal in the combined US prison and jail: Findings at 12 months post-release

## Slide 26
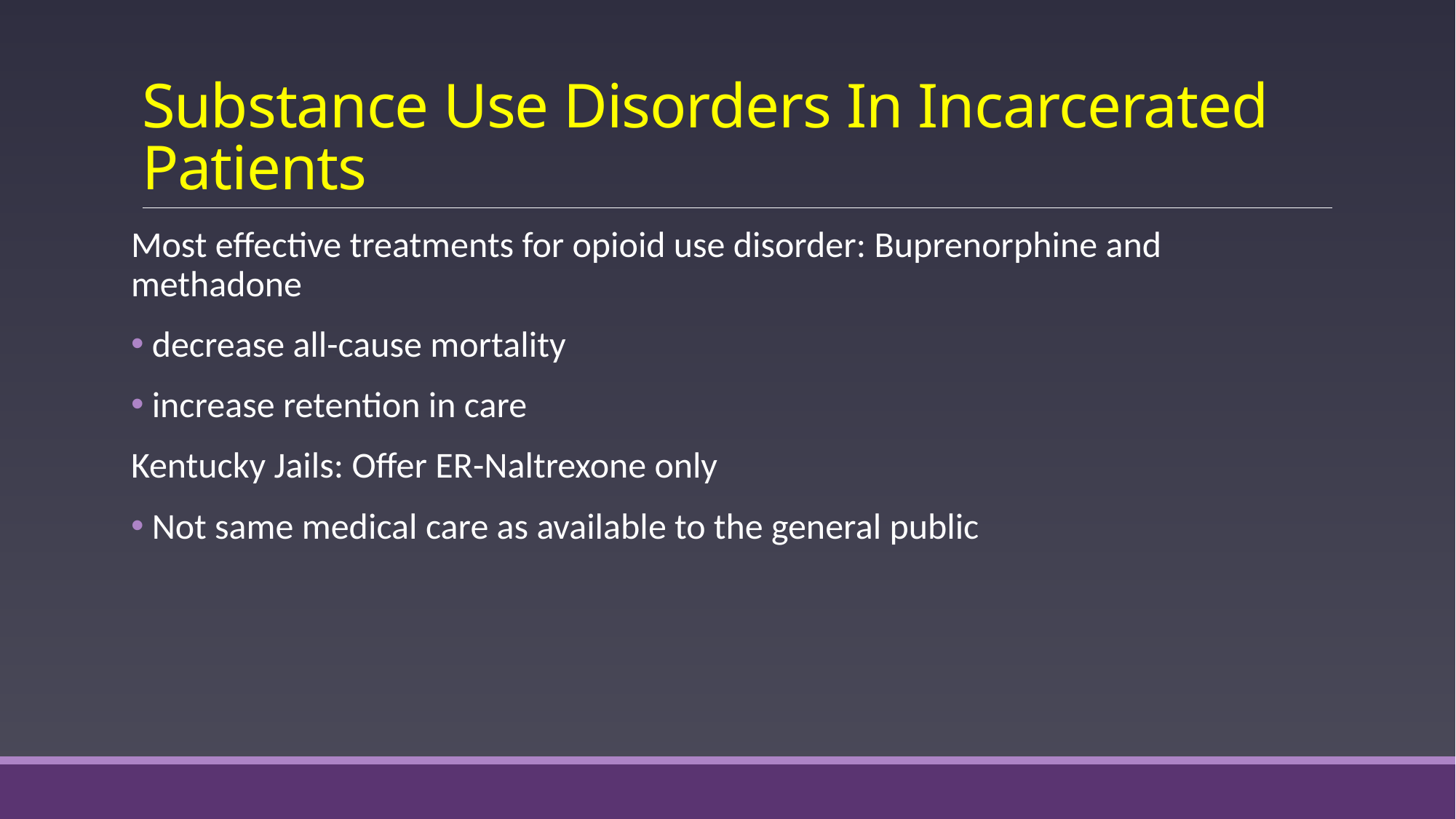

# Substance Use Disorders In Incarcerated Patients
Most effective treatments for opioid use disorder: Buprenorphine and methadone
 decrease all-cause mortality
 increase retention in care
Kentucky Jails: Offer ER-Naltrexone only
 Not same medical care as available to the general public

## Slide 27
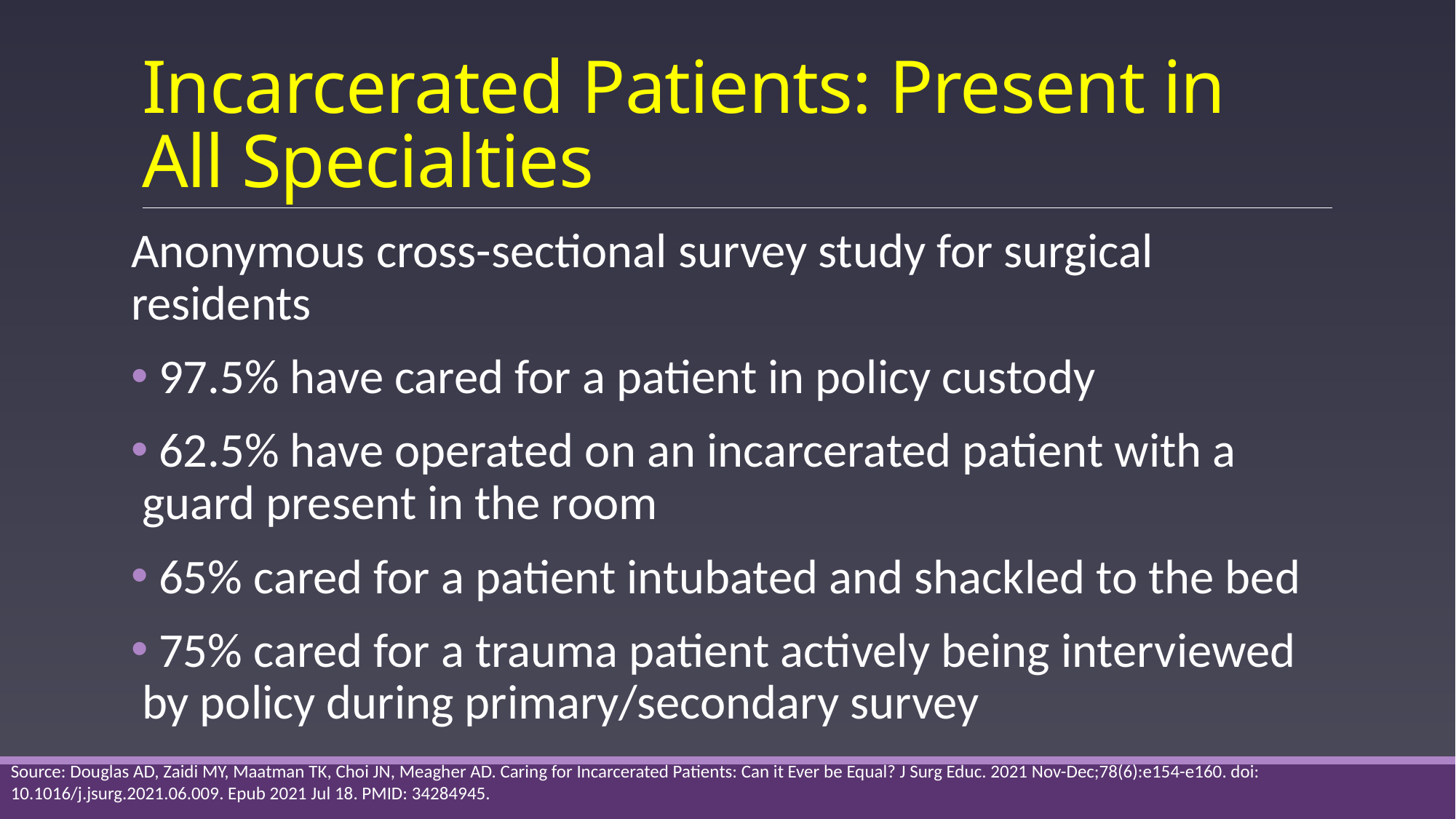

# Incarcerated Patients: Present in All Specialties
Anonymous cross-sectional survey study for surgical residents
 97.5% have cared for a patient in policy custody
 62.5% have operated on an incarcerated patient with a guard present in the room
 65% cared for a patient intubated and shackled to the bed
 75% cared for a trauma patient actively being interviewed by policy during primary/secondary survey
Source: Douglas AD, Zaidi MY, Maatman TK, Choi JN, Meagher AD. Caring for Incarcerated Patients: Can it Ever be Equal? J Surg Educ. 2021 Nov-Dec;78(6):e154-e160. doi: 10.1016/j.jsurg.2021.06.009. Epub 2021 Jul 18. PMID: 34284945.

## Slide 28
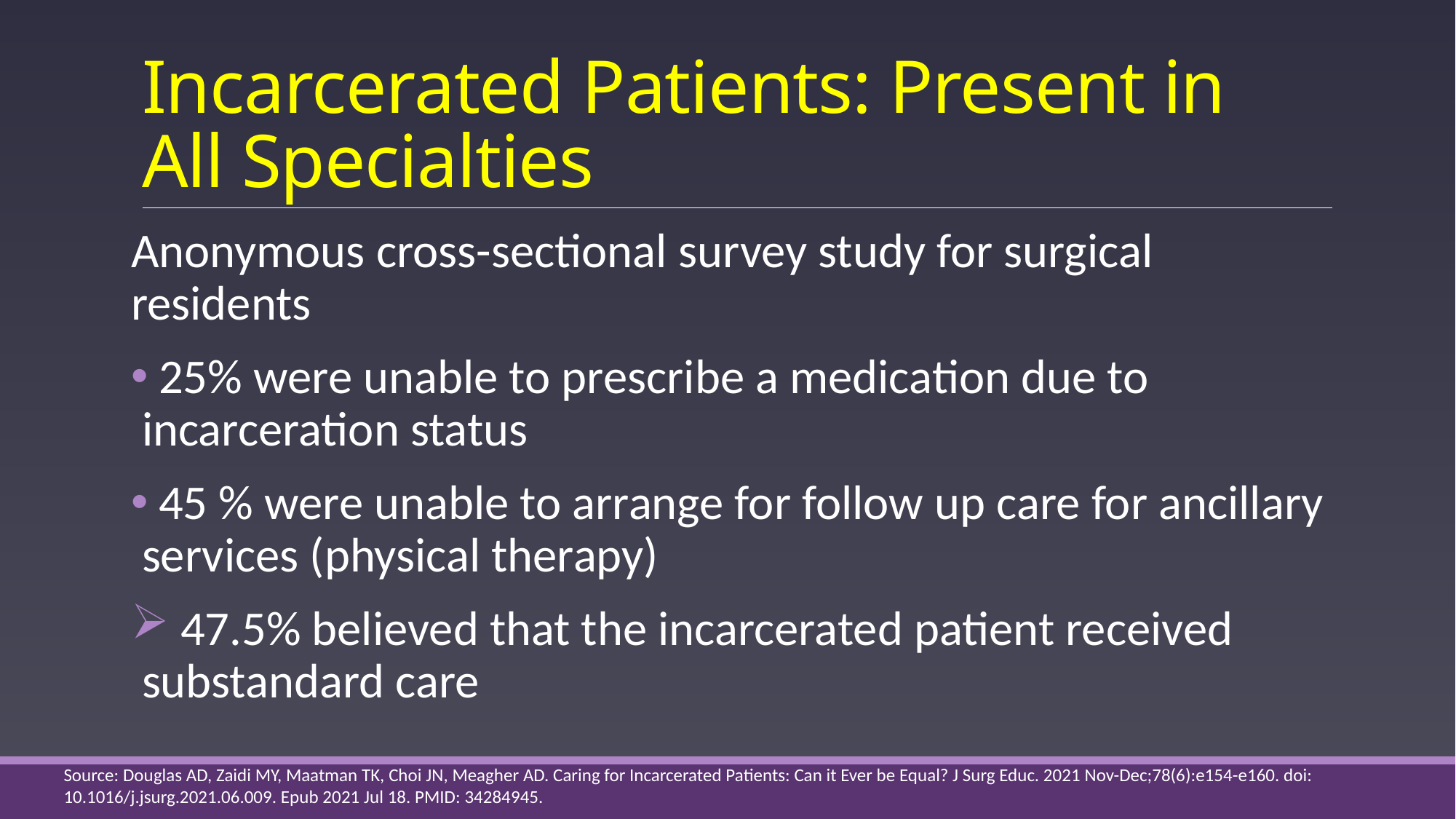

# Incarcerated Patients: Present in All Specialties
Anonymous cross-sectional survey study for surgical residents
 25% were unable to prescribe a medication due to incarceration status
 45 % were unable to arrange for follow up care for ancillary services (physical therapy)
 47.5% believed that the incarcerated patient received substandard care
Source: Douglas AD, Zaidi MY, Maatman TK, Choi JN, Meagher AD. Caring for Incarcerated Patients: Can it Ever be Equal? J Surg Educ. 2021 Nov-Dec;78(6):e154-e160. doi: 10.1016/j.jsurg.2021.06.009. Epub 2021 Jul 18. PMID: 34284945.

## Slide 29
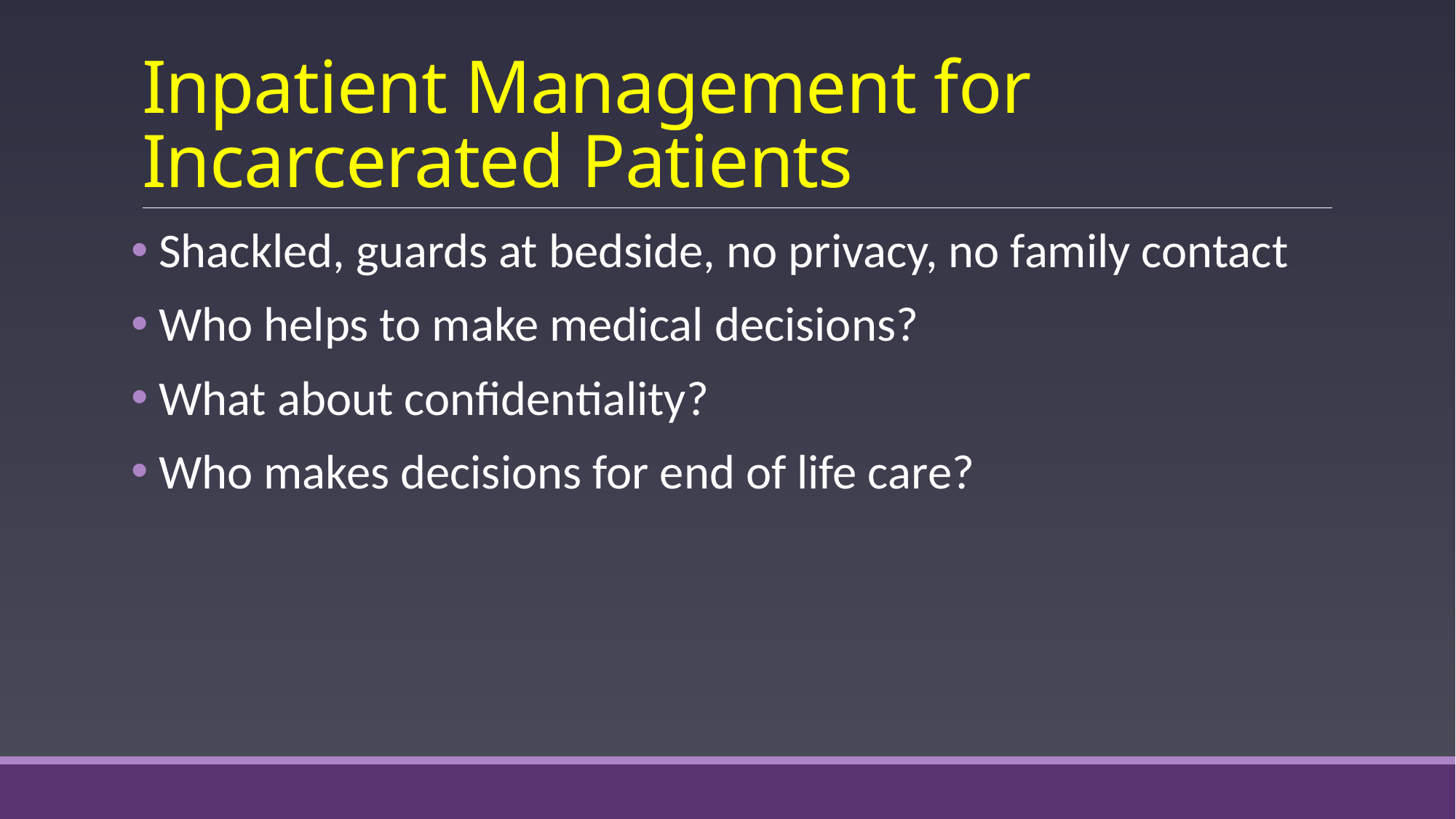

# Inpatient Management for Incarcerated Patients
 Shackled, guards at bedside, no privacy, no family contact
 Who helps to make medical decisions?
 What about confidentiality?
 Who makes decisions for end of life care?

## Slide 30
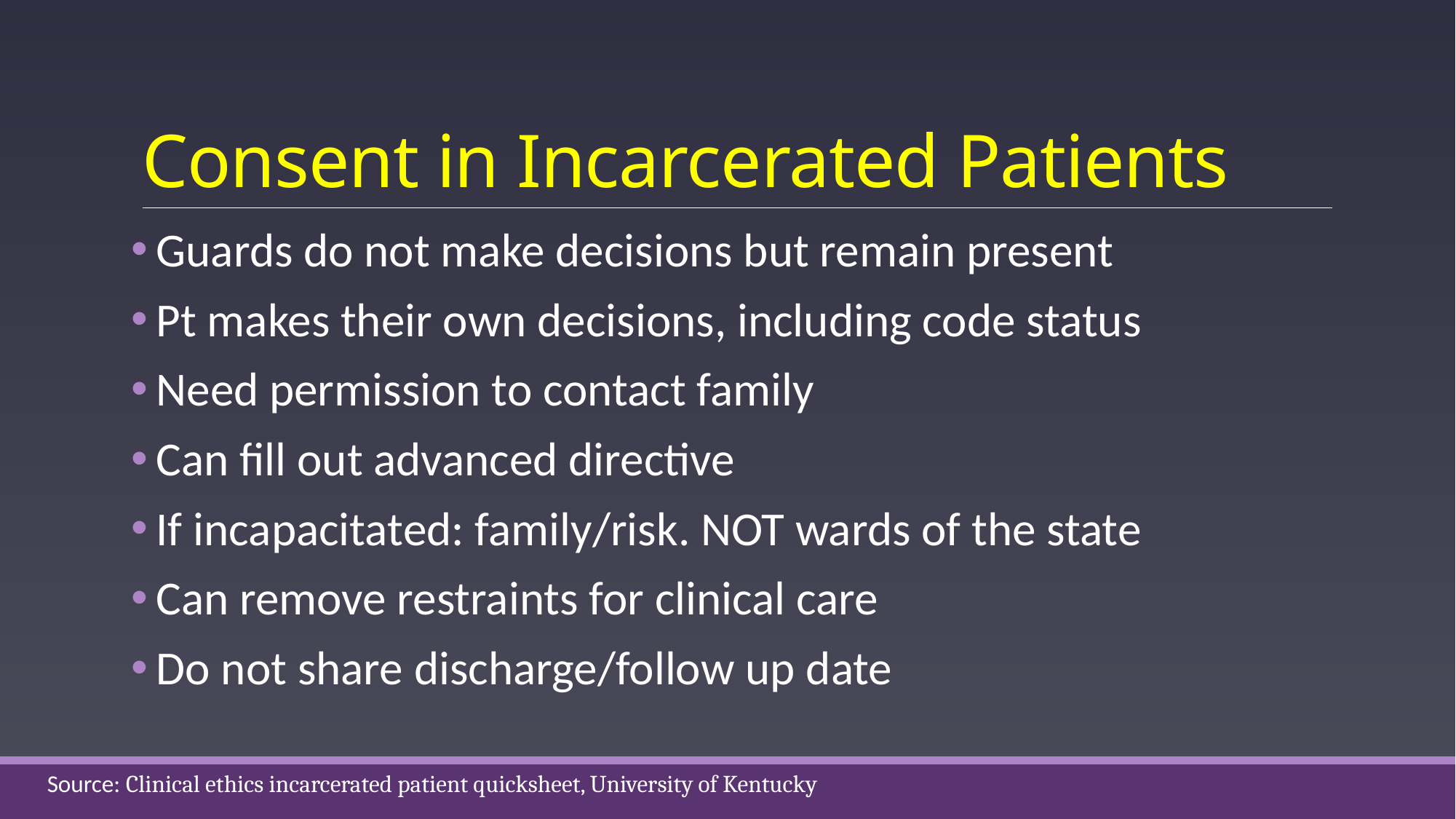

# Consent in Incarcerated Patients
 Guards do not make decisions but remain present
 Pt makes their own decisions, including code status
 Need permission to contact family
 Can fill out advanced directive
 If incapacitated: family/risk. NOT wards of the state
 Can remove restraints for clinical care
 Do not share discharge/follow up date
Source: Clinical ethics incarcerated patient quicksheet, University of Kentucky

## Slide 31
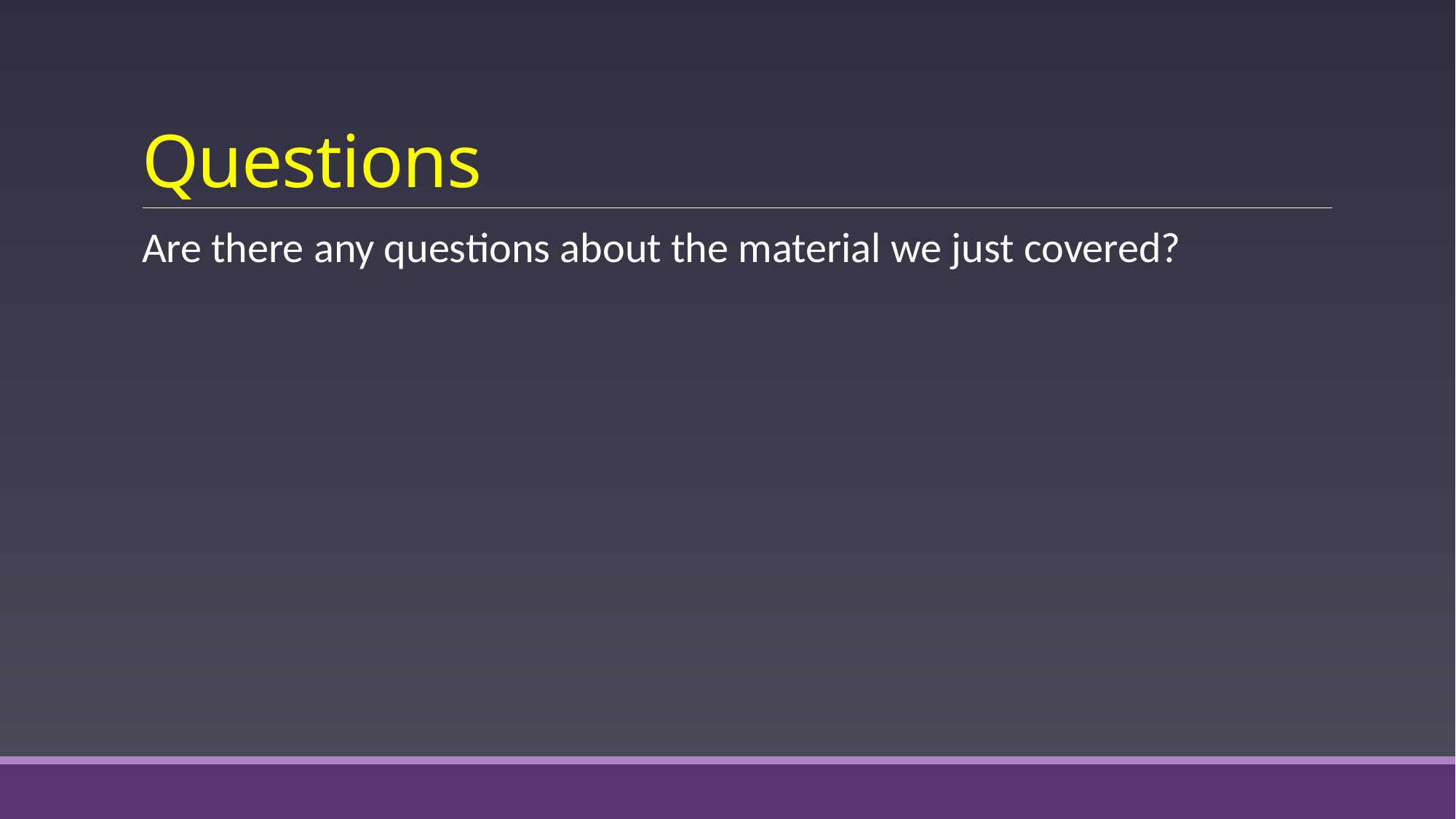

# Questions
Are there any questions about the material we just covered?

## Slide 32
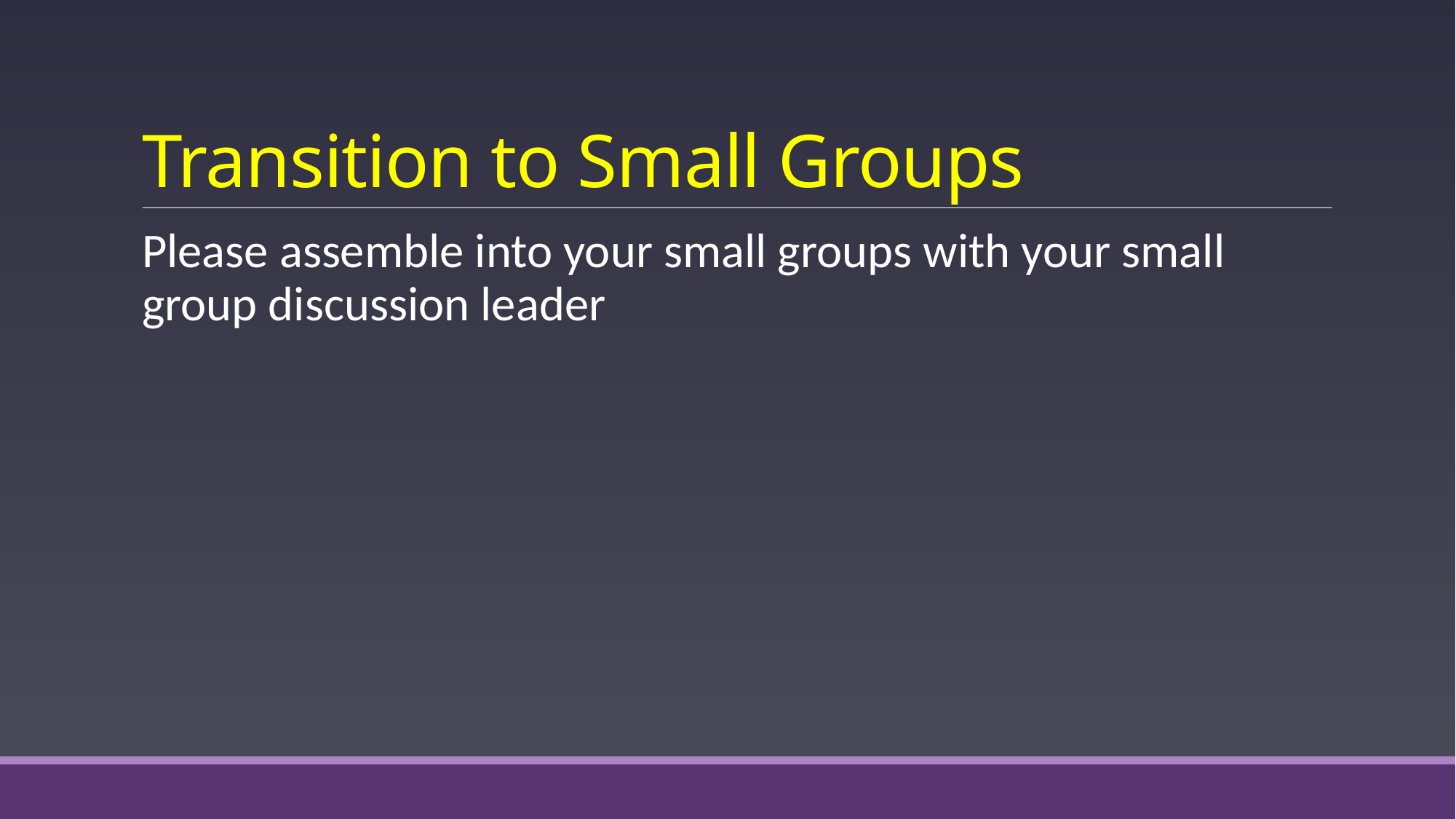

# Transition to Small Groups
Please assemble into your small groups with your small group discussion leader

## Slide 33
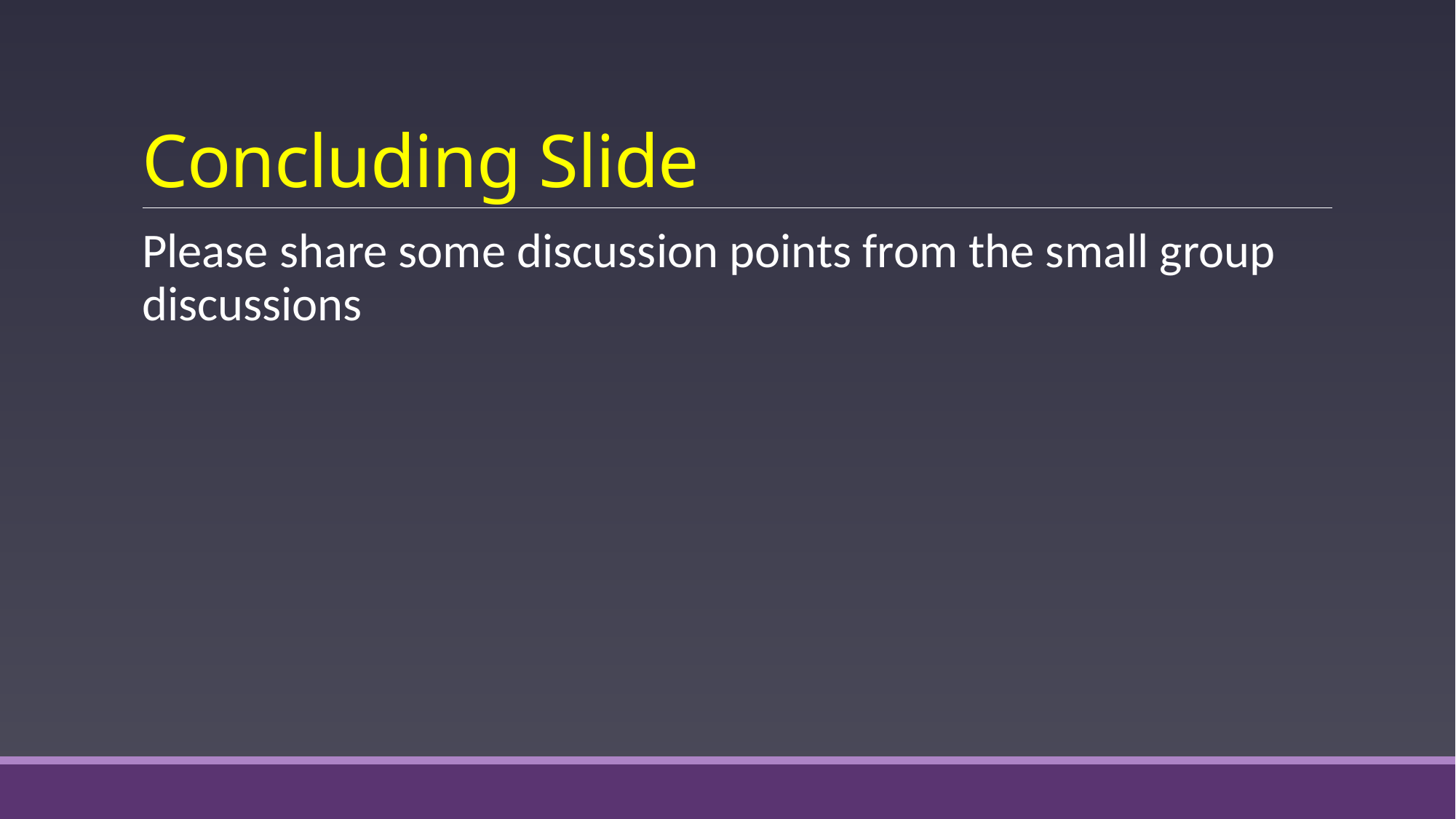

# Concluding Slide
Please share some discussion points from the small group discussions

## Slide 34
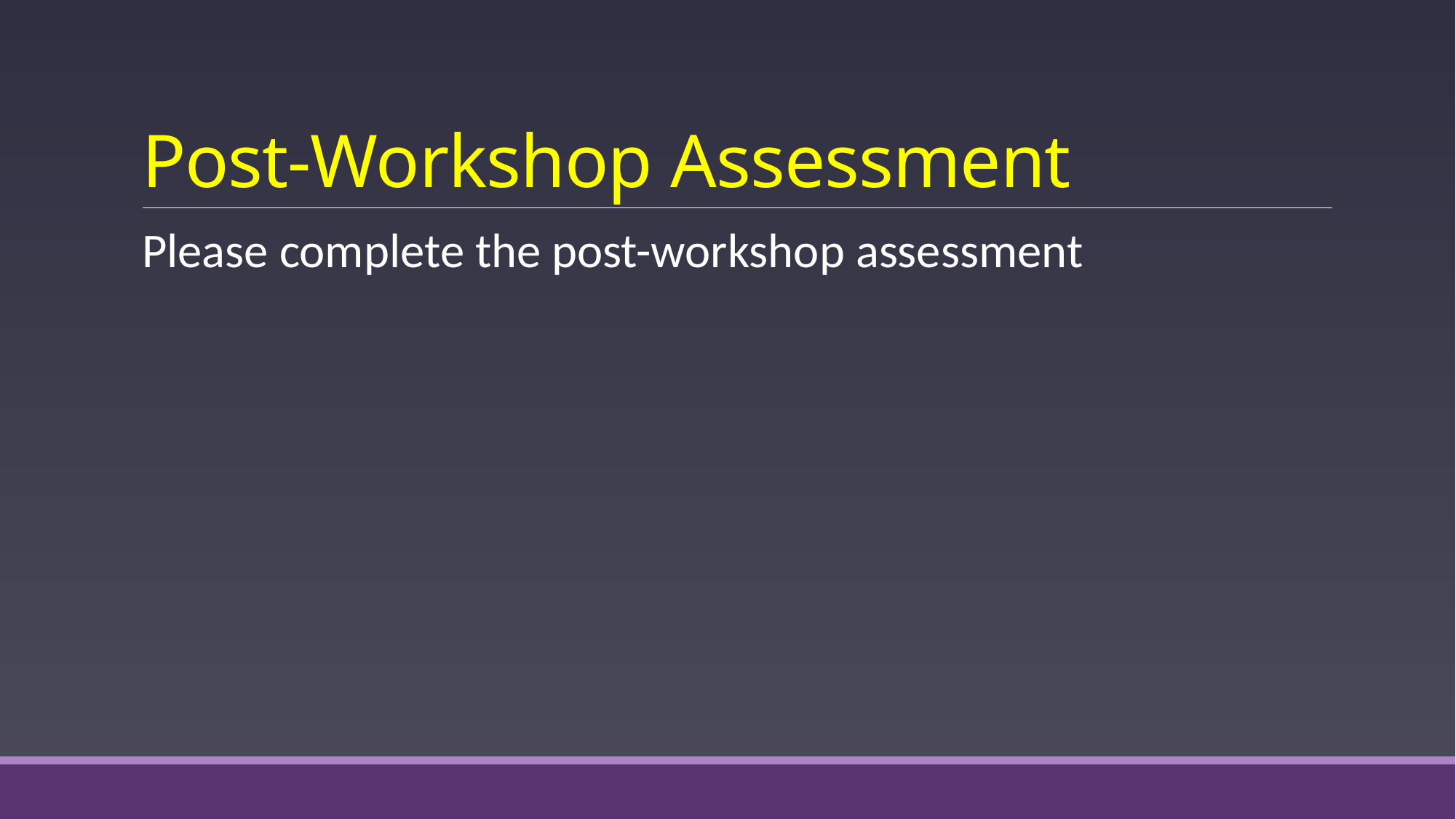

# Post-Workshop Assessment
Please complete the post-workshop assessment

## Slide 35
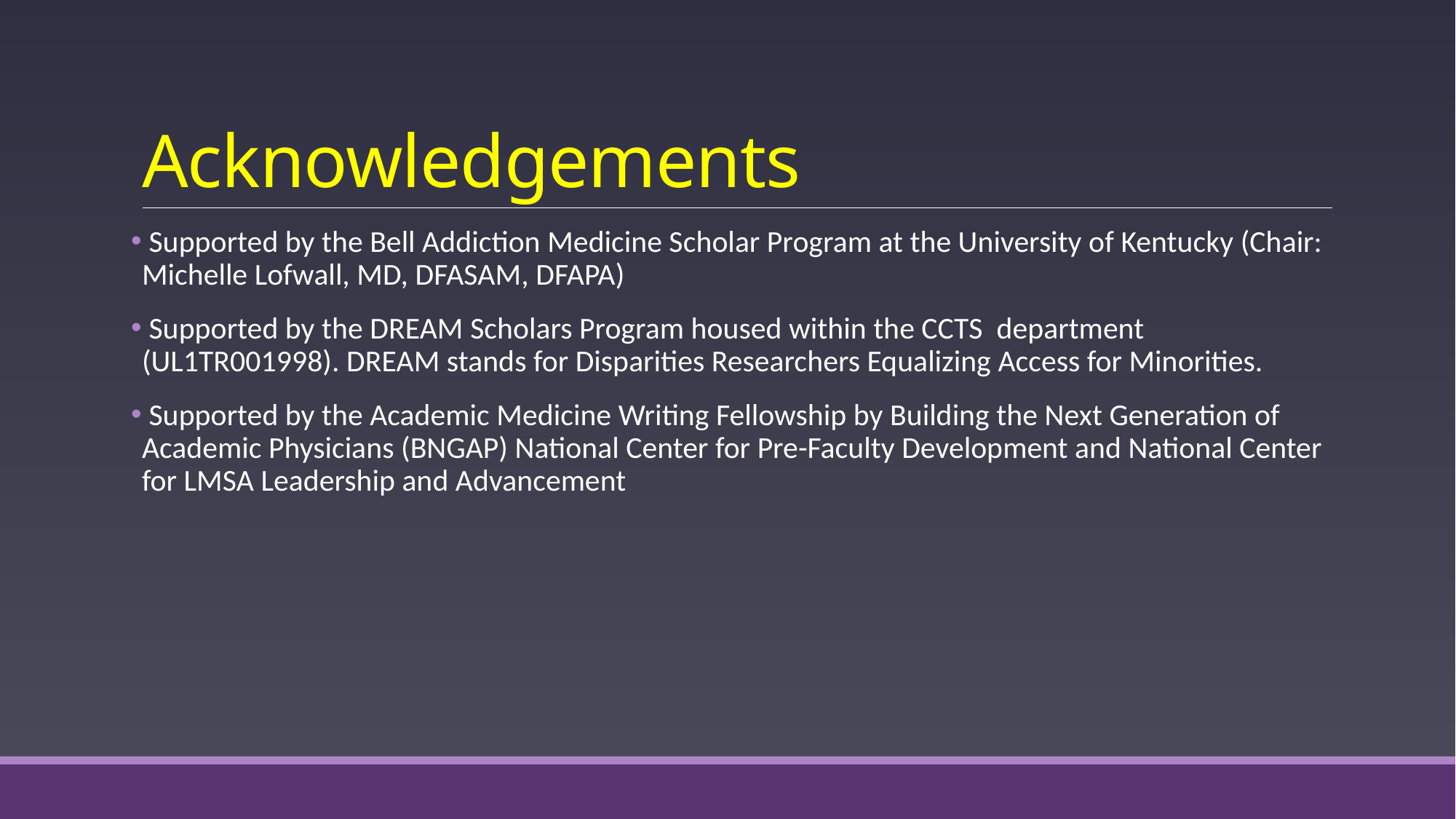

# Acknowledgements
 Supported by the Bell Addiction Medicine Scholar Program at the University of Kentucky (Chair: Michelle Lofwall, MD, DFASAM, DFAPA)
 Supported by the DREAM Scholars Program housed within the CCTS department (UL1TR001998). DREAM stands for Disparities Researchers Equalizing Access for Minorities.
 Supported by the Academic Medicine Writing Fellowship by Building the Next Generation of Academic Physicians (BNGAP) National Center for Pre-Faculty Development and National Center for LMSA Leadership and Advancement
