# Supplementary material for: Health Care for People Who Are Incarcerated: Teaching Third-Year Medical Students About Rights, Challenges, and Avenues of Advocacy
Source: MedEdPORTAL. 2024 Nov 7;20:11464. doi: 10.15766/mep_2374-8265.11464 (PMC11540842; doi:10.15766/mep_2374-8265.11464)
Supplement: Supplementary file 1 — Basics of Health Care for Incarcerated Patients.pptxFacilitator Guide.docxPretraining Session Evaluation.docxPosttraining Session Evaluation.docx [file mep_2374-8265.11464-s001.zip › B. Facilitator Guide.docx]

**Appendix B**

**The Basics of Health Care for Incarcerated Patients**

Facilitator Guide

- This is the facilitator guide to lead both the interactive large group didactic session and the small group sessions
- It accompanies the entire workshop. The workshop will last about 60 minutes
- Use this facilitator guide to deliver the interactive didactic session and then guide the small group discussion. If multiple facilitators are available for the small groups, share the relevant parts of the facilitator guide with them.

**Overall Goals:**

The goals of this facilitator guide that accompanies our workshop are to help third-year medical students understand healthcare barriers faced by people who are incarcerated, their healthcare rights, and how to advocate for equitable healthcare for this vulnerable population.

**Workshop Objectives:**

1. Describe the right to healthcare among incarcerated patients.

2. Discuss unique challenges when providing healthcare for incarcerated patients.

3. Identify avenues for physicians to advocate for equitable healthcare for incarcerated patients.

**Workshop Materials:**

Computer and projector for PowerPoint presentation

Evaluation forms for pre-and post- workshop session and pens if evaluations are done on paper

Access to a survey platform such as Qualtrics or Redcap if surveys are completed electronically

**Suggested Agenda and Timeline (approximately 60 min)**

**Part 1: 30 min, interactive didactic session with power point slides**

Pre-didactic session survey: This can be done via pen and paper or via a QR code (3-5 minutes)

This workshop serves to educate medical students on healthcare for incarcerated people. Facilitators should make themselves familiar with the slides before presenting the material. In addition, as new literature is released around this topic, these slides should be updated to reflect up-to-date data and best clinical practice.

The number of facilitators depends on the total student number in attendance and the desired size of the small groups for discussion. One facilitator is needed to present the power point slides, and one facilitator for each small group which typically has ~ 8-10 medical students. If there are not enough facilitators, students can be trained as facilitators, the small group sessions could take place in consecutive sessions with some students taking a break, the group size could be increased or the students could break into small groups and discuss the questions independently amongst themselves to then report back to the large group.

**Slide 1: Instructions**

This slide serves as an instruction slide for the facilitator of the workshop. Remove this slide before presenting the workshop.

**Slide 2: Title slide**

Add facilitator(s) name and affiliation. The facilitator(s) should introduce themselves and their roles in the institution, and can mention that they will be presenting these peer-reviewed slides that are part of today’s workshop.

**Slide 3: Pre-workshop assessment**

Add a link or code for the students to go to the pre-workshop survey assessment. Alternatively, provide papers with the pre-workshop survey assessment and pens to fill out the assessment, and collect the surveys afterwards.

**Slide 4: Learning objectives**

Read the learning objectives

**Slide 5, 6 &7:** **Case presentation of RM and interactive think, pair and share**

Tell the students that this is a fictional case

Ask one of the students to read the case of RM for their classmates: RM is a 30-year-old male who is admitted to the hospital after an assault at the local county jail. He has several facial injuries that require surgical intervention. He is shackled to the bed with one wrist and one leg. Two correction officers are present. RM would like to tell you something in private without the correction officers overhearing him. He also requests that his shackles are removed so he can be more comfortable. He asks what would happen to him if he was to not wake up after the surgery and could not make his own medical decisions.

Give students 5 min to discuss with their seat neighbor and then have each group share some of their discussion points. The discussion questions are:

- Can you ask the guards to step out so you can speak to RM privately?
- Can you remove RM’s shackles?
- Who will make decisions for RM should he not be able to make his own decisions?

Potential discussion points:

- The Health Insurance and Privacy Accountability Act (HIPAA) still applies to incarcerated patients; assess that you feel safe in the situation with the patient (they are not encephalopathic or belligerent); can ask the guards to step away as long as they maintain line of sight (stepping to the other side of the room out of ear shot, going to the other side of a door with a glass window etc.). It is best if the officers feel comfortable with where they are in regards to the patient, so the decision is amicable, but ultimately, HIPAA applies. Make sure that this is safe for you as a healthcare provider as the officers are tasked with ensuring safety which is why they need to maintain line of sight
- If it is safe for you and medically necessary, shackles can be removed. Shackles are there to address specific situations such as a safety risk or flight risk, however they can also interfere with medical care and are often not comfortable. If I need to, for example, do a walk test to assess a patient's gait, ask the guards to remove the shackles. If concern for a flight or safety risk are low enough, shared decision making between the physician and the officers should lead to shackling that is as least invasive as possible, for example just doing one ankle or removing shackles for certain periods of time. Shackles also have risks for medical complications, such as pressure ulcers and neuropathies.^1^
- It is a common misconception among clinicians that incarcerated status equates to no longer having the right to make one’s own medical decisions. People who are incarcerated have the right to make their own decisions like any other patient, unless they have an advanced directive (insert other legal document names that – e.g., health care proxy/etc). Use the same assessment to determine decision-making capacity as you do with every other patient. If they cannot make medical decisions (=do not have capacity), identify a family member or somebody that they designate as a proxy that follows the chain of decision maker for patients who do not have capacity as it is specific to individual states. In some states, this means it cannot be someone who is also incarcerated (for example, a married couple with both parties incarcerated could not designate the spouse as a medical decision maker in these states but would identify someone who is not incarcerated). The warden should not make medical decisions for patients who are incarcerated because the warden has a potential conflict of interest. There could be a security concern when reaching out to family members to make medical decisions as this reveals that the person who is incarcerated is currently in the hospital. A hospital is not a high security facility. In order to contact the decision maker for a person who is incarcerated go through the warden to then contact family. It can be helpful to get the legal department of the hospital involved and also the ethics department.^2^

**Slide 8: Current situation in the US**

Discussion Points:

- Currently in the United States, we have the highest rate of incarceration worldwide and most physicians do not get any or very limited training on how to provide health care for incarcerated patients.^2,3^
- Policies about how to navigate healthcare for this vulnerable patient population and safety for staff vary between different hospitals, between different primary care clinics, but also between different jails, different counties, different prisons, and on the state versus federal level.^4^ In addition, there is a lack of a payor source for health care for people who are incarcerated because when somebody who is on Medicaid gets incarcerated, their insurance becomes inactive.^5^

**Slide 9: Health care for incarcerated patients**

Explain that the Eighth Amendment has been interpreted by the Supreme Court to apply to healthcare for incarcerated people. It was written in 1791 not about health care, but it states that cruel or unusual punishment should not be inflicted. This was actually in reference to bond money, but the Supreme Court has applied it to medical care in multiple different rulings, most notably in 1976.^3,6^

**Slide 10: Race and gender**

Explain the demographics of incarcerated people: race and gender, male people and Black people are overrepresented.^7^

**Slide 11: Annual income of incarcerated men prior to incarceration and non-incarcerated men, ages 27-42**

Explain that people from lower socio-economic backgrounds are overrepresented in the carceral system.^8^

**Slide 12: Substance use disorder among incarcerated people**

Explain that another group that is overrepresented in the carceral system are people with substance use disorders.^9^ Sixty-five percent of people who are currently incarcerated meet DSM-5 criteria for a substance use disorder. We do not know what type of substance use disorder (e.g., alcohol use disorder or stimulant use disorder or opioid use disorder or a combination of multiple substance use disorders), which problematic as medical treatment differs for each of those substance use disorders. Only 5% receive medication for opioid use disorder, (MOUD).^9^

**Slide 13: Access to medical care (interactive)**

Ask the class if they know how a person who is incarcerated can access medical care in the carceral facility and ask them to outline how someone who is not incarcerated access health care in the community. Potential discussion points: Choices in the community about which clinician to go to and in which setting (PCP vs urgent care vs ED), and option to get a second opinion. In the carceral system, there are medical providers in each facility with certain hours, so can contact them during “sick hours” but often the first point of contact for an incarcerated person is a correction officer whose job it is to ensure security, not to provide medical care, so this is somebody that most likely does not have a medical background. The correction officer then decides if it's appropriate to reach out to a nurse. The nurse triages the patient, and then the patient is seen by a clinician who decides if you need a higher level of care outside the carceral setting, either in an emergency room or somewhere else. No option for a second opinion.

**Slide 14: Challenges to health care**

Discuss that for a lot of our patients, it can be very challenging emotionally to be diagnosed with a chronic medical disease such as hypertension, diabetes or substance use disorder, and to make the changes that are necessary to manage the chronic medical disease. Our goal as doctors is to practice shared decision making with my patients that is patient-involved to improve health literacy, overall health outcomes and give them hope that the chronic disease is not going to define their entire life. In the carceral system, the management of the disease is often very paternalistic. Medical disease can be ignored or even punished (for example, instead of providing MOUD to treat cravings and withdrawal from opioids, opioid withdrawal can be seen as part of the punishment. Incarceration has profound negative health consequences, including a reduction in life expectancy,^10^ a higher prevalence of chronic medical diseases (hypertension, asthma, cervical cancer, arthritis, and hepatitis).^11,12^ Patients will go to a medicine call once per day, where they are given a medication, for example insulin, but they are not active part of dosing their own insulin. They maybe not be taught how to count carbs, or do not have the option to do that within the food options in the carceral system.

Explain that there is a lack of federal oversight agencies for health care delivery in all the jails and prisons. There are over 5000 jails, state prisons, and federal prisons in the United States there is very little patient-involved decision making, partially due to lack of payor source.

**Slide 15: Infectious disease and incarcerated patients**

Another concern is overcrowding and frequent turnover of both people who are incarcerated and people who work in the facility. Highlight that frequently incarcerated people get transferred to other facilities and live in close quarters, resulting in high transmission of communicable airborne disease such as tuberculosis and influenza and affect both staff and incarcerated people at higher rates.^13^

**Slide 16: Challenges: Moving between systems (interactive)**

Ask the students if they have ever provided medical care for a patient who is being transferred from another hospital to them. What are the challenges that they have experienced with that? Discussion points: incomplete records including incomplete test results, medication history, imaging studies. Unclear chronology of what work-up happened when etc. or how long a patient has been receiving treatment such as antibiotics. Then ask the students to apply this to someone moving from one carceral facility to another? Potential discussion points: loss of paper work, loss of medical records, loss of medication, incomplete records or medication, changes in timing of medication administration, changes need to medication based on food that differs between different facilities (for example for patients with diabetes or heart failure). And after release from the carceral system, incarcerated patients do not get a discharge summary detailing their health care needs like one does for example at the time of hospital discharge. So, any time somebody moves from one point to another in the system, there is a risk of system breakdown, of losing their medical information, of losing their medications. All of this leads to fragmentation in medical care and potentially substandard medical care.

**Slide 17: Challenges (continued): Varying time of incarceration**

Discuss that time of incarceration varies for each individual (the average time of incarceration in state prisons is 2.7 years, compared to 28 days in county jails).^12^ Highlight that incarceration is often a tumultuous time for many incarcerated people and while 28 days might not appear to be a long time, it is especially if you are not receiving medical care for a chronic medical disease that can result in severe medical complications.

Potential examples:

- someone with heart failure could have an exacerbation due to not having access to a cardiac, low salt diet.
- someone with an opioid use disorder, who is not receiving medication for opioid use disorder or other agonist therapy, will lose their tolerance for opioids and have an increased risk of dying from an overdose after release or while in prison/jail

Highlight that there are limitations in care for psychiatric diseases.

**Slide 18: Challenges (continued): After release (interactive)**

Ask the students to name some potential challenges that an incarcerated person could face when they get released from incarceration.

Discussion points:

- If Medicaid has lapsed during incarceration, pts need to re-enroll after release
- Potential competing priorities: secure housing, employment
- Often, our patients lose their possessions while they are incarcerated, including their ID or driver's license (all necessary for signing up for insurance and other programs/making doctor appointment)
- Challenge: people may also be medically and psychiatrically ill with limited education and social support, other resources to help navigate the insurance and health care system.

**Slide 19: Mortality after release from incarceration**

Highlight that incarceration in itself is a social determinant of health with high mortality. Explain this retrospective cohort study from Washington State that looked at 30,000 people after release from incarceration (443 people died, 253 of them within one year of release).^14^ Highlight the age of the study population (18 to 44) and emphasize that the general population do not have such high rates of mortality at that age. The mortality of formerly incarcerated people is 3.5 times higher than the general population. The most common cause of death after release was death from an overdose, followed by death due to cardiovascular disease (the number one cause of death in America), homicide, and suicide. Highlight that these are all causes of death that are potentially preventable with medical intervention. The risk of death is the highest within two weeks after release, when it's 12.7 times higher than the general population. Since the number one cause of death after release from incarceration is overdose, transition to the next slides to discuss effective medication for the treatment of opioid use disorder (OUD) and opioid overdose.

**Slides 20-23: Medications for opioid use disorder (MOUD)** (interactive)

These slides are for interactive class participation and the table should be filled in together^15^:

Question to ask: What are the three Food and Drug Administration (FDA) approved medications for treatment of opioid use disorder?

Answer: Buprenorphine, methadone and naltrexone

Question: What is the mechanism of action for each of these medications?

Answer: Buprenorphine=partial opioid agonist, methadone =full opioid agonist and naltrexone = opioid antagonist

Question: Is there a difference in their effect on opioid cravings and opioid withdrawal?

Answer: Methadone and buprenorphine both substantially reduce opioid cravings and alleviate opioid withdrawal. Naltrexone diminishes opioid cravings but to a lesser extent. It does not treat opioid withdrawal.

Question: Are there differences in mortality reduction?

Answer: Buprenorphine and methadone are mortality-reducing. We do not have data showing that naltrexone is mortality reducing.

Question: Are there differences in the FDA indications for their use?

Answer: Buprenorphine and methadone are approved for treatment of opioid use disorder and naltrexone is approved for relapse prevention in opioid use disorder. Naltrexone is also FDA – approved for use in alcohol use disorder

The students may not know all these details, but the importance is to highlight that all three medications have a different mechanism of action and are therefore NOT interchangeable, as well as that methadone and buprenorphine are mortality-reducing. Discussion of MOUD would be a separate lecture in itself.

**Slide 24: Addressing the opioid overdose crisis: MOUD must be part of the solution**

This slide demonstrates the critical impact of MOUD on reducing mortality for patients with opioid use disorder in general, as well as after release from incarceration. Remind the students that the number one cause of death after release from incarceration is overdose. This slide highlights why access to MOUD is so important using standardized mortality rates.^16,17,18^ Explain to the students that this slide assigns a mortality of one to the general population, and a patient with an untreated opioid use disorder has a mortality that is more than six times higher than the general population. But if that person starts on MOUD (methadone or buprenorphine), their mortality is significantly decreased to about 1.8 times compared to the general population. This refers to all-cause mortality, not just mortality due to overdoses. Remind the students that methadone and buprenorphine have data for mortality reduction, naltrexone does not. Remind the students that people who are incarcerated have a high prevalence of substance use disorders. Explain why MOUD is so important during incarceration: We have also learned that the average time of incarceration in a county jail is 28 days. During this time, people lose their tolerance to opioids unless they receive opioid agonists (for example oxycodone) or are started on MOUD (buprenorphine and methadone) for treatment of OUD. We just also learned that the number one cause of death within two weeks after release is overdose. This is science at work. People have OUD, a chronic medical disease. They get incarcerated and no longer have access to the opioids that they were using on the street or prescribed so they are not able to maintain physical dependence on opioids. They lose their tolerance to opioids, a process that happens quickly over the matter of days and is accompanied by painful opioid withdrawal. They did not get treatment for their chronic medical disease (OUD) during their incarceration. They return to regular life after release from incarceration and have a return to opioid use with lower or no opioid tolerance, and die from an opioid overdose. These deaths are avoidable deaths if people with OUD are started (or continued) on MOUD during incarceration and are linked to follow up care after release.

**Slide 25: Methadone continuation vs. forced withdrawal**

Explain that this study from 2015 looks at patients that are on MOUD prior to incarceration and allowed patients to continue the medication for their chronic medical disease. The intervention arm here is the one where incarcerated people were allowed to stay on methadone (the standard of care for OUD) compared to the treatment as usual arm during incarceration, which forced people to discontinue methadone, the common approach of carceral settings at the time (and still occurring in many places). The study showed that the people who were allowed to continue on Methadone had a higher rate of returning to their methadone clinic within one month of release from incarceration, and had a higher engagement at 12 months after incarceration. For the treatment as usual arm, the people who were forced to discontinue methadone, a percentage of them still engaged in care for OUD after release but at a lower rate.^19,20^

**Slide 26: Substance use disorders in incarcerated patients**

Explain that while we know that buprenorphine and methadone both reduce all-cause mortality and increase retention in care, there is a discrepancy in following the best medical care and the care that is provided in carceral facilities: most only offer extended-release Naltrexone (known under the brand name Vivitrol), the only MOUD for which we do not have data that it is mortality reducing. These facilities are not following the standard of care for OUD, which is to offer all three medications to patients with OUD, so they can make a patient-centered, informed decision about which medication is best for each individual patient. In addition, they are forcing patients to discontinue their mortality-reducing medications (buprenorphine and methadone). Remind the students that this is highlighted in the article that they read and that this will be discussed this more in their small groups. The assigned article for reading highlights that OUD is a disability that is protected under the Americans with Disabilities Act (ADA). And withholding medication for OUD as a matter of policy is a violation of the ADA.

**Slide 27: Health care for incarcerated patients - present in all specialties**

Remind the students that no matter what specialty they will chose, they will be providing health care for incarcerated people. Highlight this survey of surgical residents.^21^ This is a single site study, so that has limitations for generalization. However, the survey found that 97% of surgical residents have cared for patients in police custody, 62% of them operated on an incarcerated patient with a guard present in the operating room, 65% had cared for a patient that was intubated and shackled to the bed, and 75% of them had cared for a trauma patient that was actively being interviewed by police while they were doing the primary or secondary survey in the ER (so the part where the doctors are still stabilizing the patient).^21^ There is a potential conflict of interest here. Both the police officers and the correctional officers are tasked with minimizing safety risks. Physicians are trying to provide medical care to the patient, and part of that medical care is protecting the patient’s privacy and medical information. Remind the students that this is not equitable care as it is different from care that people who are not incarcerated receive.

**Slide 28: Present in All Specialties (cont.)**

Continue to explain the findings of this study and the way the residents were forced to deliver inequitable healthcare: 25% of the residents were not able to prescribe a medication at time of discharge due to the patient being incarcerated, 45% of them were unable to arrange follow-up visits for ancillary services, specifically physical therapy and 47% of these residents interviewed in this study believed that the person that was incarcerated received substandard care compared to the patients that are not experiencing incarceration.

**Slide 29: Inpatient management for incarcerated patients (interactive)**

Ask the students if they can name any potential challenges for providing care for incarcerated patients in the hospital based on the fictional case of RM discussed at the beginning of the didactic session.

Potential discussion points: Remind them of the practice of shackling potentially interfering with medical assessments, remind them of potential breaches in privacy due to presence of correctional officers even though HIPAA still applies, and remind them that patients who are incarcerated cannot contact their family to help them with medical decision making without prior permission from the carceral system. Remind them that a lot of physicians have not received training for healthcare for incarcerated people and will therefore not know how to address these challenges.

**Slide 30: Consent in incarcerated patients**

Remind the students that another common challenge is that people do not know who will make decisions for incarcerated people and to coordinate follow up appointments. Highlight that correctional officers do not make health care decisions for people who are incarcerated, but they often remain physically present in the room and listen to doctor patient discussions. It is often feasible for officers to provide the patients privacy while doing their duty of maintaining a line of site and ensuring the patient does not escape or harm others by stepping outside the room and watching the patients through a window or if there is no window. Doing so would be in compliance with HIPAA, while they keep the patient in their line of sight. Patients who are incarcerated make their own medical decisions, including code status. They can choose to be DNR/DNI. We do need permission to contact families. When it comes to health care, incarcerated people are not considered wards of the state, meaning they do not get a decision maker assigned to them. Patients who are incarcerated can fill out an advance directive, and then if a patient is, for whatever reason, unable to make their own decisions, it follows the same decision-making tree as everybody else who is not incarcerated. However, if there are any concerns in the decision-making hierarchy for patients who are incarcerated, it is reasonable to contact the hospital lawyer and risk department for further discussion and to inquire if there are state-specific laws that need to be considered. In general, you are not supposed to share discharge plans with a patient who is incarcerated as this can be a significant safety concern for the correctional officers. This is challenging for coordination of care after hospital discharge. It is recommended work with the correctional officers in these situations to ensure that follow up recommendations and appointments are communicated with the patient, and to document it in the discharge summary, which a patient can request from the hospital after release from incarceration. The carceral setting is responsible for transporting a patient to medical follow up appointments while they are incarcerated and then the patient is aware to check their medical records after release.

**Slide 31: Opportunity for students to ask questions before they break into groups**

Ask the students if they have any questions about the material that was presented to them

**Slide 32: Transition Slide**

As the students to break into small groups to proceed to part 2 of the lecture (small group sessions), see below.

**Slide 33 (to be completed after the small group discussion):**

Come together as a large group and ask the students to share discussion highlights from their small group session

**Slide 34: Post-Workshop Evaluation**

Add a link or code for the students to go to the pre-workshop assessment. Alternatively, provide papers with the pre-workshop assessment and pens to fill out the assessment, and collect the papers afterwards.

**Slide 35: Acknowledgements**

This is an opportunity for you to make your acknowledgements. You could acknowledge MedEd Portal.

**Part 2: 30 min, small group discussion, interactive**

Divide the students into their small groups and have them sit with a facilitator

Article Discussion: Treating Opioid Use Disorder in Patients Who are Incarcerated – Quandaries of a Hospitalist^18^

<https://jamanetwork-com.ezproxy.uky.edu/journals/jama/fullarticle/2804387>

The above articles will be read by the students as prework for the session, please familiarize yourself with the article before the session. This is a perspective piece written by addiction medicine physicians who experience how a patient is prohibited from continuing mortality-reducing medication upon return to jail, significantly violating the doctors’ medical ethics and the patient’s rights. The article provides some resources for advocacy for this vulnerable patient population. Here are some potential facilitator questions:

1. What are some potential violations of medical ethics that you can identify in this viewpoint? Highlight each as outlined below
   - beneficence (Do what is good for the patient. The patient wants medication, does not want to continue in substance use. He has a chronic medical disease (OUD) and deserves to receive the standard of care which is MOUD because it treats withdrawal, cravings and reduces his mortality.)
   - non-maleficence (do no harm. Withholding MOUD that is mortality reducing is harmful. The patient experienced direct harm as substance use continued after release and he had to return to the hospital multiple times and has high risk of mortality)
   - justice (as physicians we need to advocate for just allocation of resources. Withholding it from an entire patient group is not just.)
   - autonomy (the patient wants to make his own medical decisions and has a right to chose MOUD or not and if he choses MOUD, has a right to choose which kind. Forcing him to discontinue buprenorphine violates his autonomy.)
2. What rights does this patient have? (Remind the students that the Eight Amendment has been interpreted by the Supreme Court as applicable to health care, so he has a right to health care, and withholding health care would be considered cruel and unusual punishment. He also has a disability (OUD) and it is illegal to discriminate against him for being on MOUD under the Americans with Disabilities Act (ADA). Discontinuing buprenorphine as a blanket policy without an individualized medical assessment to determine if he benefits from being on it is a violation of the ADA.)
3. What are some potential negative health outcomes that this patient could suffer due to his incarceration? (Remind the students of the high risk of mortality after release from incarceration, with overdose being the number one cause of death. Remind them that buprenorphine decreases all cause mortality and he in general has an increased mortality from untreated OUD, including infections, trauma and suicide. Also highlight that there was no continuity of care as he was not transported to follow up appointments, even though this was arranged during his hospitalization.)
4. How would his care have differed if he was not incarcerated? (Remind the students that the patient would have access to a second opinion to assess if he should continue buprenorphine or not. He would have been allowed to choose between the sublingual product or the long acting injectable and would have been linked to outpatient follow up for his ODU. In addition, he would have had other options for treatment of OUD such as residential treatment in addition to medication.)
5. What are some potential avenues for advocacy? (Discuss that as a first step, a clinician should talk to their counterparts in the carceral facility to see what medical care patients can receive at that facility and if they cannot receive the required care, should inquire about transferring a patient to a different facility. In addition, one can educate the patient on his rights under the Eighth Amendment and the ADA, recommend to get legal representation. Both the medical providers and the patient can report ADA violation to the assistant US attorney and the other sources listed in the article. A last resort option would be to refuse to discharge the patient from the hospital until a safe plan for MOUD can be established)

References:

1. Haber LA, O’Brien M. Shackling Ulcer: an Upper Extremity Ulcer Secondary to Handcuffs. *J Gen Intern Med*. 2021;36(7):2146-2146. doi:https://doi.org/10.1007/s11606-021-06654-3
2. Haber LA, Erickson HP, Ranji SR, Ortiz GM, Pratt LA. Acute Care for Patients Who Are Incarcerated: A Review. *JAMA Intern Med*. 2019 Nov 1;179(11):1561-1567. doi: 10.1001/jamainternmed.2019.3881. PMID: 31524937.
3. Alsan M, Yang CS, Jolin JR, Tu L, Rich JD. Health Care in U.S. Correctional Facilities – A Limited and Threatened Constitutional Right. *New England Journal of Medicine*. 2023; 388 (9): 847-852.
4. Haber LA, Kaiksow FA, Williams BA, Crane JT. Hospital care while incarcerated: A tale of two policies. *Journal of Hospital Medicine*. 2024 Mar; 19(3):230-234 . <https://doi.org/10.1002/jhm.13223>
5. Wakeman SE, McKinney ME, Rich JD. Filling the gap: the importance of Medicaid continuity for former inmates. *J Gen Intern Med*. 2009 Jul;24(7):860-2. doi: 10.1007/s11606-009-0977-x.
6. Eber GB Using the Constitution to Improve Prisoner Health. *Am J Public Health*. 2009. 99(9). 1541-1542.
7. Federal Bureau of Prisons. BOP statistics: Inmate race. Bop.gov. Published November 11, 2023. https://www.bop.gov/about/statistics/statistics_inmate_race.jsp
8. National Institute on Drug Abuse. Criminal Justice DrugFacts. National Institute on Drug Abuse. Published June 1, 2020. https://nida.nih.gov/publications/drugfacts/criminal-justice#:~:text=There%20are%20high%20rates%20of
9. ‌Berg MT, Rogers EM, Lei MK, Simons RL. Losing Years Doing Time: Incarceration Exposure and Accelerated Biological Aging among African American Adults. *J Health Soc Behav*. 2021;62(4):460-476. doi:10.1177/00221465211052568
10. Binswanger IA, Krueger PM, Steiner JF. Prevalence of chronic medical conditions among jail and prison inmates in the USA compared with the general population. *J Epidemiol Community Health*. 2009;63(11):912-919. doi:10.1136/jech.2009.090662
11. Kendig NE, Butkus R, Mathew S, Hilden D; Health and Public Policy Committee of the American College of Physicians. Health Care During Incarceration: A Policy Position Paper From the American College of Physicians. *Ann Intern Med*. 2022 Dec;175(12):1742-1745. doi: 10.7326/M22-2370. Epub 2022 Nov 22. PMID: 36410006.
12. Restum ZG. Public health implications of substandard correctional health care. *Am J Public Health*. 2005 Oct;95(10):1689-91. doi: 10.2105/AJPH.2004.055053. PMID: 16186448; PMCID: PMC1449420.
13. Binswanger IA, Stern MF, Deyo RA, Heagerty PJ, Cheadle A, Elmore JG, Koepsell TD. Release from Prison – A High Risk of Death of Former Inmates. *N Engl J Med*. 2007. 356 (2). 157-165
14. Substance Abuse and Mental Health Services Administration. Medications for Opioid Use Disorder. Treatment Improvement Protocol (TIP) Series 63 Publication No. PEP21-02-01-002. Rockville, MD: Substance Abuse and Mental Health Services Administration, 2021
15. Dupouy J, Palmaro A, Fatseas M, Auriacombe M, Micallef J, Oustric S, Lapeyre-Mestre M. Mortality Associated With Time In and Out of Buprenorphine Treatment in French Office-Based General Practice: A 7-year Cohort Study. *Ann Fam Med*. 2017. 15(4): 355-358.
16. Evans E, Li L, Min J, Huang D, Urada D, Liu L, Hser YI, Nosyk B. Mortality Among Individuals Accessing Pharmacological Treatment for Opioid Use Disorder in California, 2006-2010. *Addiction*. 2015. 110 (6): 996-1005.
17. Sordo L, Barrio G, Bravo MJ, Indave BI, Degenhardt L, Wiessing L, Ferri M, Pastor-Barriuso R. Mortality Risk During and After Opioid Substitution Treatment: Systemic Review and Meta-Analysis of Cohort Studies. *BMJ*. 2017. 357:j1550.
18. Rich JD, McKenzie M, Larney S, Wong JB, Tran L, Clarke J, Noska A, Reddy M, Zaller N. Methadone continuation versus forced withdrawal on incarceration in a combined US prison and jail: a randomized, open-label trial. *Lancet*. 2015. 386. 350-359.
19. Brinkley-Rubinstein L, McKenzie M, Macmadu A, Larney S, Zaller N, Dauria E, Rich JD. A randomized, open label trial for methadone continuation versus forced withdrawal in the combined US prison and jail: Findings at 12 months post-release. *Drug Alcohol Depend.* 2018 Mar 1:184:57-63. doi: 10.1016/j.drugalcdep.2017.11.023.
20. Douglas AD, Zaidi MY, Maatman TK, Choi JN, Meagher AD. Caring for Incarcerated Patients: Can it Ever be Equal? *J Surg Educ*. 2021 Nov-Dec;78(6):e154-e160. doi: 10.1016/j.jsurg.2021.06.009.
21. South AM, Fanucchi LC, Lofwall MR. Treating Opioid Use Disorder in Patients Who Are Incarcerated: Quandaries of a Hospitalist. *JAMA*. 2023. 329(20), 1738–1739. https://doi.org/10.1001/jama.2023.5904
