# Supplementary material for: Health Care for People Who Are Incarcerated: Teaching Third-Year Medical Students About Rights, Challenges, and Avenues of Advocacy
Source: MedEdPORTAL. 2024 Nov 7;20:11464. doi: 10.15766/mep_2374-8265.11464 (PMC11540842; doi:10.15766/mep_2374-8265.11464)
Supplement: Supplementary file 1 — Basics of Health Care for Incarcerated Patients.pptxFacilitator Guide.docxPretraining Session Evaluation.docxPosttraining Session Evaluation.docx [file mep_2374-8265.11464-s001.zip › C. Pretraining Session Evaluation.docx]

**Appendix C**

- This is the pre-training survey that students should complete before the workshop
- It will take about 3-5 minutes
- Use this pre-training survey at the appropriate slide (Appendix A) during the interactive didactic session.

**Pre-Training Survey**

**Identifier:** Create a unique identifier (for example, a random letter and four random numbers)

**Demographics:**

1. What is your gender?
2. Woman
3. Man
4. Genderqueer
5. Non-Binary
6. Not Listed: ___________
7. Prefer not to reply
8. Are you transgender or cisgender (i.e. not transgender)?
   1. Cisgender
   2. Transgender
   3. Prefer not to reply
9. Are you Hispanic, Latino or of Spanish origin?
   1. Yes
   2. No
   3. Prefer not to answer
10. How do you identify yourself?
    1. American Indian or Alaska Native
    2. Asian
    3. Black or African American
    4. Native Hawaiian or Other Pacific Islander
    5. White
    6. Prefer not to answer

**Perception Questions:**

1. Prior to this lecture, I have received adequate training on providing health care for incarcerated patients
2. True
3. False
4. Unsure
5. It is important for physicians to advocate for patients who are incarcerated to ensure the receive adequate medical care
6. Extremely important
7. Important
8. Neutral
9. Low importance
10. Not at all important
11. I feel confident that I know how to provide care for incarcerated patients
12. Strongly agree
13. Agree
14. Neither agree nor disagree
15. Disagree
16. Strongly disagree

**Knowledge Questions:**

1. Patients who are incarcerated have a constitutional right to health care
2. True
3. False
4. Unsure
5. Incarceration is a social determinant of health as evidenced by the finding that incarcerated persons have a higher risk for mortality than someone who is not incarcerated
6. True
7. False
8. Unsure
