# Supplementary material for: Health Care for People Who Are Incarcerated: Teaching Third-Year Medical Students About Rights, Challenges, and Avenues of Advocacy
Source: MedEdPORTAL. 2024 Nov 7;20:11464. doi: 10.15766/mep_2374-8265.11464 (PMC11540842; doi:10.15766/mep_2374-8265.11464)
Supplement: Supplementary file 1 — Basics of Health Care for Incarcerated Patients.pptxFacilitator Guide.docxPretraining Session Evaluation.docxPosttraining Session Evaluation.docx [file mep_2374-8265.11464-s001.zip › D. Posttraining Session Evaluation.docx]

**Appendix D**

- This is the post-training survey that students should complete after the workshop
- It will take about 3-5 minutes
- Use this post-training survey at the appropriate slide (Appendix A) during the interactive didactic session.

**Post-Training Survey**

**Identifier:** Create a unique identifier (for example, a random letter and four random numbers)

**Perception Questions:**

1. This lecture has significantly increased my knowledge about health care for incarcerated patients
2. Strongly agree
3. Agree
4. Neutral
5. Disagree
6. Strongly disagree
7. It is important for physicians to advocate for patients who are incarcerated to ensure the receive adequate medical care
8. Extremely important
9. Important
10. Neutral
11. Low importance
12. Not at all important
13. I feel confident that I know how to provide care for incarcerated patients
14. Strongly agree
15. Agree
16. Neither agree nor disagree
17. Disagree
18. Strongly disagree

**Knowledge Questions:**

1. Patients who are incarcerated have a constitutional right to health care
2. True
3. False
4. Unsure
5. Incarceration is a social determinant of health as evidenced by the finding that incarcerated persons have a higher risk for mortality than someone who is not incarcerated
6. True
7. False
8. Unsure

**Free Text Feedback Questions:**

What did learn from this workshop?

How would you improve this workshop?
